# Self-assembled cyanidin-3-O-glucoside nanoparticles alleviate inflammation and ferroptosis induced by PRRSV infection

**DOI:** 10.1128/jvi.00954-25

**Published:** 2025-08-14

**Authors:** Xiaohan Chen, Yipeng Pang, Fructueux Modeste Amona, Zilu Liu, Fang Wang, Yuan Liang, Jiachen Yang, Wanhan Zhang, Xingtang Fang, Xi Chen

**Affiliations:** 1 Institute of Cellular and Molecular Biology, School of Life Science, Jiangsu Normal University 12675, Xuzhou, Jiangsu, China; Loyola University Chicago - Health Sciences Campus, Maywood, Illinois, USA

**Keywords:** self-assembly, cyanidin-3-O-glucoside, PRRSV, ferroptosis, reactive oxygen species, anti-inflammation

## Abstract

Porcine reproductive and respiratory syndrome virus (PRRSV) is a highly contagious pathogen that causes substantial economic losses in the global swine industry, primarily by impairing reproductive performance and respiratory health. However, current therapeutic approaches remain limited in effectively controlling PRRSV infection. Here, we present self-assembled cyanidin-3-O-glucoside (C3G)-based nanoparticles (C3G-Cs-SeNPs) as a promising antiviral agent against PRRSV infection. These nanoparticles demonstrated excellent stability and biocompatibility, with minimal cytotoxicity in Marc-145 cells. C3G-Cs-SeNPs significantly reduced ROS levels in PRRSV-infected cells, enhancing the antioxidant capacity and mitigating oxidative stress. In addition, they effectively inhibited key stages of the PRRSV lifecycle, including viral internalization and replication, leading to a marked decrease in viral proliferation. Moreover, C3G-Cs-SeNPs alleviated ferroptosis by restoring redox homeostasis through enhanced antioxidant enzyme activity. Mechanistically, these nanoparticles potentially activate the SIRT1/Nrf2/HO-1 signaling pathway, which plays a critical role in regulating inflammation and ferroptosis in PRRSV-infected cells. These findings suggest that C3G-Cs-SeNPs could serve as a novel therapeutic approach to modulate inflammation, ferroptosis, and viral replication in respiratory viral infections. Our study highlights the therapeutic potential of C3G-Cs-SeNPs in combating PRRSV infection, with important implications for viral infection management and respiratory disease treatment.

**IMPORTANCE:**

Porcine reproductive and respiratory syndrome virus (PRRSV) remains a major challenge in the swine industry, causing significant economic losses due to its high mutation rate and ability to evade host immunity. Current antiviral treatments and vaccines offer limited efficacy, necessitating the development of novel therapeutic strategies. This study introduces self-assembled cyanidin-3-O-glucoside-based chitosan-selenium nanoparticles (C3G-Cs-SeNPs) as a promising antiviral agent. These nanoparticles effectively inhibit PRRSV replication, reduce oxidative stress, and alleviate inflammation and ferroptosis by activating the SIRT1/Nrf2/HO-1 signaling pathway. By mitigating virus-induced cellular damage, C3G-Cs-SeNPs offer a potential therapeutic approach for PRRSV and other respiratory viral infections. This study highlights the role of ferroptosis in PRRSV pathogenesis and presents an innovative nanotechnology-based solution to combat viral infections, contributing to the development of more effective antiviral strategies.

## INTRODUCTION

Porcine reproductive and respiratory syndrome virus (PRRSV) is a highly contagious virus that leads to substantial economic losses in the global swine industry, primarily owing to its impact on reproductive performance and respiratory health (1). Its rapid mutation and evasion of the host immune response make PRRSV particularly challenging to control (2).

Ferroptosis, an iron-dependent form of regulated cell death, results in the accumulation of lipid peroxides, which causes cellular damage and tissue injury, particularly in the lungs during acute respiratory infections (3–6). This process contributes to inflammation through oxidative stress, further aggravating lung injury, impairing tissue repair, and promoting persistent viral infections (7). The interplay between ferroptosis and inflammation creates a vicious cycle that hinders effective infection resolution and increases susceptibility to secondary infections, thus contributing to the chronicity of PRRSV infection. While oxidative stress and ferroptosis have been well studied in various diseases (8, 9), limited research has focused on viral infections. Therefore, targeting the host mechanisms involved in ferroptosis, inflammation, and oxidative stress offers a promising strategy to mitigate viral-induced damage and support long-term recovery.

Although several vaccines and antiviral drugs have been developed (10–12), their efficacy in controlling PRRSV infection and reducing tissue damage remains limited. Natural products, particularly anthocyanins like cyanidin-3-O-glucoside (C3G), have garnered attention for their broad-spectrum antiviral, antioxidant, and anti-inflammatory properties. Recent studies have shown that C3G can inhibit ferroptosis and reduce organ injury by enhancing oxidative resistance and boosting glutathione (GSH) levels (13, 14), a key factor in viral pathogenesis. In addition, C3G has shown protective effects against respiratory viruses, including RSV, HSV-1, and SARS-CoV-2, by alleviating oxidative stress and inflammation, making it a potential candidate for treating respiratory viral infections like PRRSV (15). However, the clinical use of C3G is limited by its poor solubility, low absorption, and limited bioavailability (16, 17). These challenges have prompted the development of advanced drug delivery systems, such as nanoparticles, to improve the solubility and bioavailability of C3G, thus enhancing its therapeutic potential (17–19).

Selenium nanoparticles (SeNPs) have demonstrated antiviral, antioxidant, and anti-inflammatory properties, proving effective against a range of viral infections, including PRRSV (20, 21). SeNPs can mitigate cell injury induced by ferroptosis-related conditions, such as cadmium exposure (22). However, their clinical application is limited by poor stability in aqueous solutions and potential toxicity at high doses. To address these issues, modifications like coating SeNPs with chitosan (Cs) have been shown to enhance their stability, cellular uptake, and antioxidant activity in aqueous environments (23, 24). Recent studies have also demonstrated that Cs-SeNPs can reduce PRRSV-induced apoptosis in Marc-145 cells by modulating the ROS/JNK signaling pathway, thereby inhibiting PRRSV replication (21). This suggests that self-assembled C3G-based Cs-SeNPs could be an effective approach for treating viral infections like PRRSV. While SeNPs-based nanoparticles like PEG-SeNPs and ZnO-SeNPs have shown potential antiviral activity (25–27), their clinical use is limited by issues such as aggregation in physiological conditions and cytotoxicity at high doses. By contrast, the self-assembled C3G-Cs-SeNPs in this study combine C3G with chitosan-coated SeNPs, harnessing the antioxidant effects of C3G and the stabilizing properties of chitosan. This enhances nanoparticle stability in aqueous environments and improves biocompatibility. In addition, C3G-Cs-SeNPs offer dual functions, targeting viral replication and host oxidative stress pathways, providing a significant advantage over traditional SeNPs that mainly act through direct antiviral mechanisms.

In this study, we investigated the potential of C3G-Cs-SeNPs to mitigate inflammation and ferroptosis during PRRSV infection. Characterization of C3G-Cs-SeNPs showed spherical particles with an average size range of 180–240 nm and a zeta potential of −11 mV. These nanoparticles demonstrated significant antioxidant activity, effectively neutralizing ROS generated during PRRSV infection. We further found that C3G-Cs-SeNPs significantly inhibited key stages of the PRRSV lifecycle, including viral internalization and replication, resulting in decreased viral proliferation. Moreover, C3G-Cs-SeNPs influenced the expression of pro-inflammatory factors in infected cells, alleviating oxidative stress and ferroptosis by enhancing redox homeostasis and decreasing ROS and lipid peroxidation. Interestingly, C3G-Cs-SeNPs activated the SIRT1/Nrf2/HO-1 pathway, aiding in regulating inflammation and ferroptosis in PRRSV-infected cells. Thus, C3G-Cs-SeNPs could be a promising therapeutic candidate for preventing and treating inflammation and ferroptosis related to PRRSV infection.

## MATERIALS AND METHODS

### Reagents, cells, and virus

Na_2_SeO_3_ (10,641C) was purchased from Adamas (Shanghai, China). Cyanidin-3-O-glucoside (C137683) was purchased from Aladdin (Shanghai, China). Chitosan (C6340) was purchased from Macklin (Shanghai, China). Other general qualitative analysis-grade chemicals were purchased from Aladdin (China) without additional purification. The SOD/MDA/GSH/CAT/MPO determination kits were purchased from Solarbio (BC5165/BC0025/BC1175/BC0205/BC5715, Beijing, China). Pro-inflammatory cytokines (IL-1β, IL-6, and TNF-α) ELISA kits were purchased from Elabscience (E-EL-M0037, E-EL-M0044, E-EL-M3063, Wuhan, China).

Marc-145 (African green monkey kidney epithelial cell line) and rPAMs (recombinant porcine alveolar macrophages, immortalized cell line derived from primary PAMs) were maintained at 37°C and 5% CO_2_ in Dulbecco modified Eagle medium (DMEM) and RPMI 1640 supplemented with 10% fetal bovine serum (FBS; GIBCO) containing 100 U/mL penicillin and 100 µg/mL streptomycin.

The highly pathogenic (HP) PRRSV strain BB0907 (GenBank accession no. HQ315835.1) (28), the classical PRRSV S1 strain (C-PRRSV) (GenBank accession no. AF090173) (29, 30), and PRRSV FJ1402 (GenBank accession no. KX169191.1); all the strains were independently inoculated into Marc-145 cells and rPAMs. The inoculation was done at a multiplicity of infection (MOI of 1 or using the amounts indicated in the figure legends or results.

### Preparation of C3G-Cs-SeNPs

The self-assembly of C3G-Cs-SeNPs was achieved by sequentially mixing the components. Briefly, 1.5 mL chitosan (10 mg/mL) was mixed with an equivalent volume of 0.1 M Na_2_SeO_3_, then ultrapure water was added with a constant volume of up to 10 mL and stirred for 30 min to form a homogeneous mixture. Subsequently, 5 mL of freshly prepared C3G (6.7 mg/mL) was added sequentially, then the mixed solution was stirred for an additional 1 hour. The self-assembly of the nanoparticles occurred spontaneously due to the interactions between the chitosan, C3G, and selenium, forming a stable C3G-Cs-SeNPs nanocomposite. The Cs-SeNPs were prepared by adding 0.4 M ascorbic acid in place of C3G, using the same methods mentioned above. The products were purified through centrifugation at 12,000 rpm for 15 minutes, followed by three washes with deionized water to eliminate any remaining reactants. The purified Cs-SeNPs and C3G-Cs-SeNPs were then freeze-dried to obtain a dry powder for further characterization.

### Characterization of C3G-Cs-SeNPs

The synthesized Cs-SeNPs and C3G-Cs-SeNPs were thoroughly characterized to assess their size, morphology, chemical composition, and stability. Scanning electron microscopy (SEM; Zeiss sigma 300) and transmission electron microscopy (TEM; JEOL F200) were utilized to provide high-resolution images of the surface morphology and size distribution of the nanoparticles or their internal structure. X-ray photoelectron spectroscopy (XPS; Thermo Fisher Scientific, K-Alpha) was carried out to confirm the presence of selenium (Se) in its reduced form, and X-ray diffraction (XRD; D8 Advance, Bruker) analysis to observe the crystalline Se. Ultraviolet-visible (UV-Vis) spectroscopy was used to analyze spectrograms, and dynamic light scattering (DLS) provided information on hydrodynamic diameter and size distribution, with zeta potential (Malvern, ZetaSizer Nano ZS) measurements conducted to assess NPs stability in suspension.

### Stability of SeNPs in different solvents

C3G-Cs-SeNPs were dispersed in deionized water, 0.9% NaCl, PBS, and DMEM at a concentration of 0.5 mg/mL, then sonicated for 15 min to evaluate their stability. DLS monitored particle size distribution over 12 hours, observing aggregation or sedimentation. The long-term stability of the dispersions was evaluated by storing the dispersions at 4°C.

### Measurement of ABTS and DPPH radical scavenging *in vitro*

The antioxidant capabilities of the C3G, Cs-SeNPs, and C3G-Cs-SeNPs were evaluated based on their capacity to scavenge ABTS free radicals (ABTS+•), DPPH free radicals (DPPH•), H_2_O_2_, and hydroxyl free radicals (•OH). The scavenging efficacy of C3G, Cs-SeNPs, and C3G-Cs-SeNPs on these free radicals was determined as previously reported (31). To prepare ABTS+•, a 1:1 combination of 7.4 mM ABTS and 2.6 mM (NH_4_)_2_S_2_O_8_ was produced and held at 4°C in the dark overnight. Then, 100 µL of ABTS+• working solution (0.37 mM) was combined with 100 µL of various C3G, Cs-SeNPs, and C3G-Cs-SeNPs doses. The UV absorbance at 734 nm was measured at various intervals to assess the C3G, Cs-SeNPs, and C3G-Cs-SeNPs’ capacity to scavenge ABTS+•. Finally, varied doses of C3G, Cs-SeNPs, and C3G-Cs-SeNPs were combined with a DPPH• ethanol solution (125 µmol mL^−1^, 100 µL) at a 1:1 ratio and incubated at 37°C for 15 min to determine the scavenging efficacy of C3G, Cs-SeNPs, and C3G-Cs-SeNPs against DPPH• using UV absorbance at 517 nm at different intervals.

### Measurement of hydroxyl radical scavenging *in vitro*

The Fenton reaction produced •OH by combining 100 µL of 32 mM FeCl_3_⋅6H_2_O, 5% hydroxylamine hydrochloride, and 100 µL of 300 mM H_2_O_2_. Methylene blue trihydrate (MB, 100 µL, 60 µg/mL) was used to collect the resulting •OH. Following that, different quantities of C3G-Cs-SeNPs were added to the •OH-containing solution, and the absorbance values at 664 nm were measured with a spectrophotometer to determine C3G, Cs-SeNPs, and C3G-Cs-SeNPs’ capacity to scavenge •OH.

### Measurement of H_2_O_2_ scavenging *in vitro*

The ability of C3G, Cs-SeNPs, and C3G-Cs-SeNPs to scavenge H_2_O_2_ was determined using an H_2_O_2_ detection kit. H_2_O_2_ interacts with ammonium molybdate to produce a persistent yellow complex with an absorbance peak at 405 nm. Different quantities of C3G, Cs-SeNPs, and C3G-Cs-SeNPs were incubated with H_2_O_2_ (2 mM) at 37°C for 24 hours. Following the reaction, the residual H_2_O_2_ concentration was evaluated according to the manufacturer’s instructions, and the ability of C3G, Cs-SeNPs, and C3G-Cs-SeNPs to eliminate H_2_O_2_ was estimated.

### Cytotoxicity assay

Marc-145 cells were seeded into 96-well plates at 1 × 10^4^ cells/well density and grown to 80%–90% confluence. They were subsequently incubated with C3G, Cs-SeNPs, and C3G-Cs-SeNPs at various concentrations (0, 5, 10, 15, 20, 25, 30, 40, and 60 µg/mL). Cells fed in DMEM served as controls. Following independent incubations for 24, 36, and 48 hours, the standard CCK8 assay assessed cell viability as previously described (21).

### Antiviral activity assay

The cells were incubated with the pretreated PRRSV at an MOI of 0.1 for 1 hour. Afterward, the inocula were discarded, and cells were washed three times with PBS and incubated with C3G, Cs-SeNPs, and C3G-Cs-SeNPs at different concentrations (0, 7.5, 15, and 30 µg/mL) for an additional 24 hours at 37°C. The samples were subsequently used for virus titration, indirect immunofluorescence assay (IFA), quantitative real-time (qRT)-PCR, and Western blotting analysis.

### Virus absorption assay

Marc-145 cells were treated with DMEM containing PRRSV and either C3G-Cs-SeNPs or control at 4 °C for 2 hours to promote virus binding. Cells were washed three times with PBS to remove any unbound virus particles and chemicals, and a fresh medium was added. The cells were then incubated at 37 °C for 46 h, and samples were subsequently used for virus titration, indirect immunofluorescence assay, qRT-PCR, and Western blotting.

### Virus internalization assay

Marc-145 cells were treated with an essential medium containing PRRSV at 4 °C for 2 hours, which promotes virus binding but not internalization. After three PBS washes, cells were transferred to a fresh medium and grown at 37 °C to promote viral internalization. C3G-Cs-SeNPs were added at 0 hours and removed after 3 hours of incubation at 37 °C. Cells were washed three times with PBS to remove free virus particles and chemicals and incubated for 43 hours at 37 °C in a fresh medium. The relative expression of viral level (expressed as a fold change) was analyzed by virus titration, indirect immunofluorescence assay, qRT-PCR, and Western blotting.

### Viral RNA replication and release assay

Marc-145 cells were infected with PRRSV at 37 °C for 6 hours and then washed three times with PBS to remove free virus particles. Fresh medium containing C3G-Cs-SeNPs was added, and cells were harvested 3 hours later for virus titration, indirect immunofluorescence assay, qRT-PCR, and Western blotting. In further investigation, Marc-145 cells were infected with PRRSV at 37 °C for 2 hours and then grown in fresh medium for 24 h. Cells were treated in a fresh medium containing C3G-Cs-SeNPs, and at 2 hours post-switch, both cell supernatants and cells were harvested for RT-PCR analysis.

### Virus titration

Marc-145 cells grown in 96-well plates at a density of 1 ×  10^4^ cells/well were infected with 10-fold serial dilutions of PRRSV. After 1 hour at 37°C, the culture medium was supplemented with fresh DMEM containing 2% FBS. Viral titrations were measured using endpoint dilution analysis 5 days post-inoculation (dpi), and the Reed–Muench method was used to calculate the 50% tissue culture infectious dose (TCID_50_) as previously reported (21, 32).

### Indirect IFA

Pretreated Marc-145 cells were fixed with precooled methanol at 4°C for 10 min and subsequently permeabilized with 0.5% Triton X-100 for 10 min. The cells were washed three times with PBS before being treated with 10% bovine serum albumin (BSA) at 4°C and incubated with the primary antibody for 12 h. Subsequently, the cells were treated with the secondary antibody and incubated at 37°C for 2 hours. The cells’ nuclei were stained with 4′, 6′-diamidino-2-phenylindole (DAPI, Invitrogen, China) for 5 min and washed with PBS. Fluorescent images were acquired with a fluorescence microscope (Leica DM4000 B).

### qRT PCR

Total RNA was extracted from cell supernatants using the Qiagen RNeasy kit (Qiagen, Hilden, Germany), and complementary DNA (cDNA) was obtained using an RT-qPCR kit (TaKaRa, Kusatsu, Japan). Quantitative real-time PCR was performed using the SYBR Premix Ex TaqTM II (Tli RNaseH Plus, TaKaRa). Relative gene expression levels were computed using the 2^−ΔΔCt^ method, normalized to GAPDH, and compared to the control group. The primer sequences used are shown in (Table 1).

**TABLE 1:** Primer sequences for the relative real-time PCR assay^*a*^

Primer	Nucleotide sequence (5′−3′)
mGAPDH-F	CCTTCCGTGTCCCTACTGCCAA
mGAPDH-R	GACGCCTGCTTCACCACCTTCT
mNrf2-F	ATTCAATGATTCTGACTCTG
mNrf2-R	CGTATCCCCAGAAGAATGTA
PRRSV-N-F	AAACCAGTCCAGAGGCAAG
PRRSV-N-R	TCAGTCGCAAGAGGGAAAT
mIL-1β-F	TGGCCCTAAACAGATGAAGT
mIL-1β-R	GGGAACCAGCATCTTCCTTA
mIL-6-F	CTGCCTTCCCCGCCCCAGTA
mIL-6-R	ATGTTACTCCTGTTACATGT
mTNF-α-F	AGTCAGATCATCTTCTCGAA
mTNF-α-R	TTCTGATGGCACCACCAGCT
mCOX2-F	CAACACTCACAACAAAACTA
mCOX2-R	TTATTATACGGACTGGGGCT
mSLC7A11-F	CTGGGTGGAACTGCTCGTAA
mSLC7A11-R	GTTCTGCGTTTGACCTTTAA
mKeap1-F	CCCTGGAGGATCATACCAAG
mKeap1-R	ATGCCCTCGATGGACACCAC
mFTH1-F	GCCAGATCAACCTGGAGCTC
mFTH1-R	TGGTTTCTTGATATCCTGAA
mGPX4-F	GCATCGTCACCAACGTGGCC
mGPX4-R	TGCCCTTGGGCTGGATCTTC
mSIRT1-F	AGCATCTTGCCTGATTTGTA
mSIRT1-R	ACAATGAGAAGATCAACTTC
mNrf2-F	GAAGTAGGTAACTGTAGTCC
mNrf2-R	AGCTTTGCAAAGTGATAGAT
mHO-1-F	GAGCGCTAGAGGAAGAGCTG
mHO-1-R	GAAGATTCGGACTGTTCTTG
mNQO1-F	GAACTTTGCCAAATACTTTCTTCAC
mNQO1-R	GCAGTCAGCCCATTCTCCC
pIL-1β-F	CCCAAAACCATCAAAGATGA
pIL-1β-R	GACTTCGCTCCCTCTCTTAC
pIL-6-F	CTGCCTTCCCTACCCCGGGA
pIL-6-R	TTCTCATACTTCTCACACAT
pTNF-α-F	GTCGCAGGAGCCACCACGCT
pTNF-α-R	GTGGGCGACGGGCTTATCTG
pCOX2-F	AATGGCTGCAGAGTTAGAAG
pCOX2-R	CCCATAAGTCCTTTCAAGGA
pSLC7A11-F	GGATCTTCATCTCTCCTAAG
pSLC7A11-R	TAGTGAAGTAGGCCACATTT
pKeap1-F	GTCATGAATGGTGCTGTCAT
pKeap1-R	CTCAGACTCGCAGCGCACAT
pFTH1-F	AGAACTTTGCCAAATACTTT
pFTH1-R	TGTGTGACTTCATTGAGACG
pGPX4-F	CCCGCGACGACTGGCGATGT
pGPX4-R	GGGCATCGTCCCCATTCACA
pSIRT1-F	CATGAAGTATGACAAAGATG
pSIRT1-R	CCACCTAATCTATGACACAA
pNrf2-F	AACACAGATTTTGGTGATGA
pNrf2-R	GATTCCACTGAGTGTTCTGG
pHO-1-F	GAGGAGATTGAGCACAACAA
pHO-1-R	CTGAGCAATCTTCTTGAGGA
pNQO1-F	GAACTTTGCCAAATACTTTCTTCAC
pNQO1-R	GCAGTCAGCCCATTCTCCC

^*a*^ m means monkey, p means pig.

### Western blotting analysis

Protein samples were separated by 10% sodium dodecyl sulfate-polyacrylamide gel electrophoresis (SDS-PAGE) and analyzed using Western blotting to measure the protein levels and phosphorylated forms of endogenous proteins as previously described (21). After the membranes were blocked with 5% low-fat milk for 2 hours at room temperature, the samples were incubated with the primary antibodies including IL-1β (1:1,000; 16806-1-AP, Proteintech), IL-6 (1:1,000; 21865-1-AP, Proteintech), TNF-α (1:1,000; 17590-1-AP, Proteintech), COX2 (1:1,000; 27308-1-AP, Proteintech), Keap1 (1:1,000; 10503-2-AP, Proteintech), SLC7A11 (1:1,000; 26864-1-AP, Proteintech), FTH1 (1:1,000; 11682-1-AP, Proteintech), GPX4 (1:1,000; ET1706-45, Huaan), SIRT1 (1:1,000; 13161-1-AP, Proteintech), Nrf2 (1:1,000; T55136, Abmart), HO-1 (1:1,000; 10701-1-AP, Proteintech), NQO1 (1:1,000; T56710, Abmart), GAPDH (1:1,000; P30008, Abmart), and N protein (1:1,000; GTX637947, GeneTex). Then, samples were incubated with secondary antibodies, goat anti-rabbit or goat anti-mouse IgG conjugated (1:1,000; Abcam, MA, USA) with horseradish peroxidase. A system based on enhanced chemiluminescence (ECL) was used to visualize immunoreactive proteins (Beyotime, P0018M). All experiments were repeated at least three times to ensure result reliability.

### ROS generation assay

Cellular ROS were detected by oxidizing the non-fluorescent DCFH into green fluorescent DCF using the dichlorofluorescein diacetate (DCF-DA) assay kit (Solarbio, China) based on previously described methods with modifications (21, 25). Marc-145 cells were infected with PRRSV at an MOI of 0.1 for 1 hour and then treated with C3G-Cs-SeNPs for 24 hours. Afterward, the cells were incubated with 10 µM DCFDA at 37°C for 30 min to detect ROS. Cells were washed with PBS and imaged using an inverted fluorescence microscope to visualize the fluorescence intensity, which indicates intracellular ROS levels.

### Mitochondrial membrane potential assay

The mitochondrial membrane potential (ΔΨm) was assessed using the lipophilic fluorescent dye JC-1 (MedChemExpress, HY-K0601) as previously described (33). Marc-145 cells were seeded in a 24-well plate at a 5 ×  10^4^ cells/well density and infected with PRRSV. After 24 hours, the cells were treated with 250 µL fresh medium containing 2.5 µL JC-1 (final concentration of 2 µM) for 20 min at 37°C. Carbonyl cyanide m-chlorophenyl hydrazone (CCCP) was used as a positive control, with 0.25 µL CCCP added to the cells to reach a final concentration of 50 µM. The cells were incubated for an additional 5 min at 37°C and washed three times with 1 × PBS. Cells were harvested for subsequent immunofluorescence analysis to assess alterations in mitochondrial membrane potential.

### Intracellular lipid peroxidation assay

The lipid peroxidation assay was performed as previously described (34). Proteins were extracted from Marc-145 cells using RIPA lysis buffer, and their concentration was measured using the BCA Protein Assay Kit according to the manufacturer’s protocol. The levels of key biomarkers of oxidative stress, including catalase (CAT), superoxide dismutase (SOD), malondialdehyde (MDA), glutathione (GSH), and myeloperoxidase (MPO) in the cell supernatant were measured according to the manufacturer’s instructions for each respective enzyme activity.

### Statistical analysis

Data were analyzed using SPSS statistical software (version 21.0), and results were reported as mean ± SD, while graphs were made with GraphPad Prism 8.3. Statistical significance was established by one-way ANOVA and LSD post hoc test, with *P <* 0.05 considered statistically significant.

## RESULTS AND DISCUSSION

### Preparation and characterization of C3G-Cs-SeNPs

**Fig 1:**
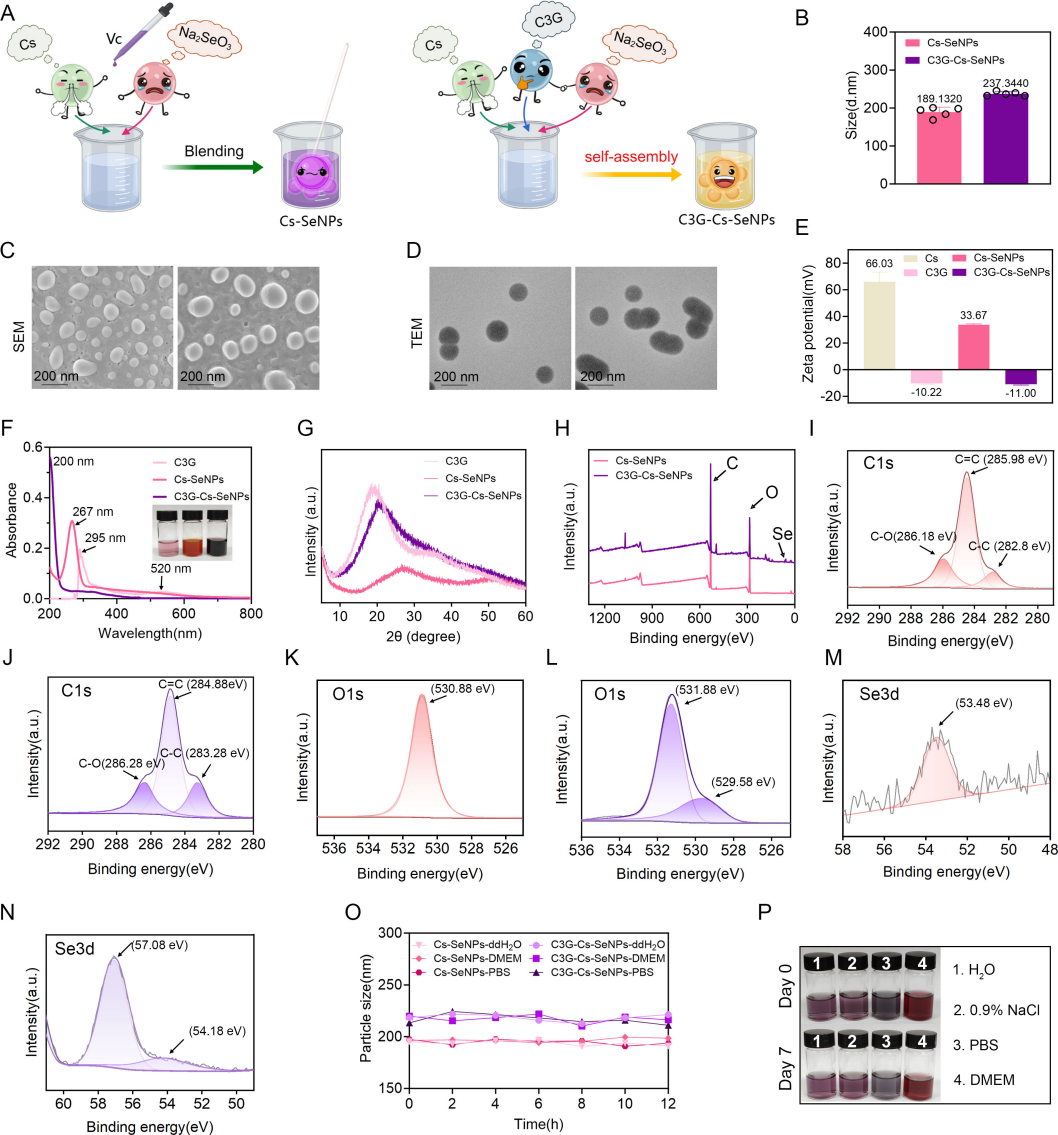
Synthesis and characterization of C3G-Cs-SeNPs. (**A**) Schematic diagram of Cs-SeNPs and C3G-Cs-SeNPs self-assembly synthesis steps. (**B**) Size distribution of Cs-SeNPs and C3G-Cs-SeNPs. (**C**) SEM (scale bar = 200 nm) and (**D**) TEM image (scale bar = 100 nm) of Cs-SeNPs and C3G-Cs-SeNPs. (**E**) Zeta potential of the C3G, Cs, Cs-SeNPs, and C3G-Cs-SeNPs. (**F**) UV-Vis spectroscopy of C3G (deionized water, pH ~7.0), Cs-SeNPs, and C3G-Cs-SeNPs. (**G**) XRD spectrum of C3G, Cs-SeNPs, and C3G-Cs-SeNPs. (**H**) Total XPS patterns of Cs-SeNPs and C3G-Cs-SeNPs. (**I through N**) High-resolution of C 1s (**I and J**), O 1s (**K and L**), and Se (**M and N**) peaks in XPS of Cs-SeNPs and C3G-Cs-SeNPs. (**O**) Hydrodynamic sizes of Cs-SeNPs and C3G-Cs-SeNP in different solutions during storage measured by DLS (*n* = 3). (**P**) Dispersibility and stability of C3G-Cs-SeNPs in H_2_O, 0.9% NaCl, FBS, and DMEM on days 0 and 7.

Cs-SeNPs and C3G-Cs-SeNPs were successfully synthesized by a self-assembly process (Fig. 1A), as previously described (23, 35). DLS results revealed that the size distribution values of the Cs-SeNPs and C3G-Cs-SeNPs are 189.1320 and 237.3440 nm, respectively (Fig. 1B). SEM further confirmed the spherical morphology and size distribution of the nanoparticles, while TEM provided high-resolution images, revealing spherical particles with an average size range of 180–240 nm (Fig. 1C and D), indicating the stability of the obtained nanoparticles. This stability is illustrated in Fig. 1E, showing the zeta potential of Cs, C3G, Cs-SeNPs, and C3G-Cs-SeNPs at 66.03 mV, −10.22 mV, 33.67 mV, and −11 mV, respectively, which indicates the successful preparation and adequate stability of C3G-Cs-SeNPs. In comparison, a similar study reported that the prepared OSAS-SeNPs and FA-OSAS-SeNPs were spherical and showed good dispersibility, with an average size of 104.84 ± 0.11 nm and 131.66 ± 7.87 nm, respectively (35). In addition, CS-SeNPs were reported to have an average diameter of 90 ± 13 nm (21). Together, these comparisons highlight that the composition of the natural compounds used in synthesizing SeNPs significantly impacts the size of NPs.

**Fig 2:**
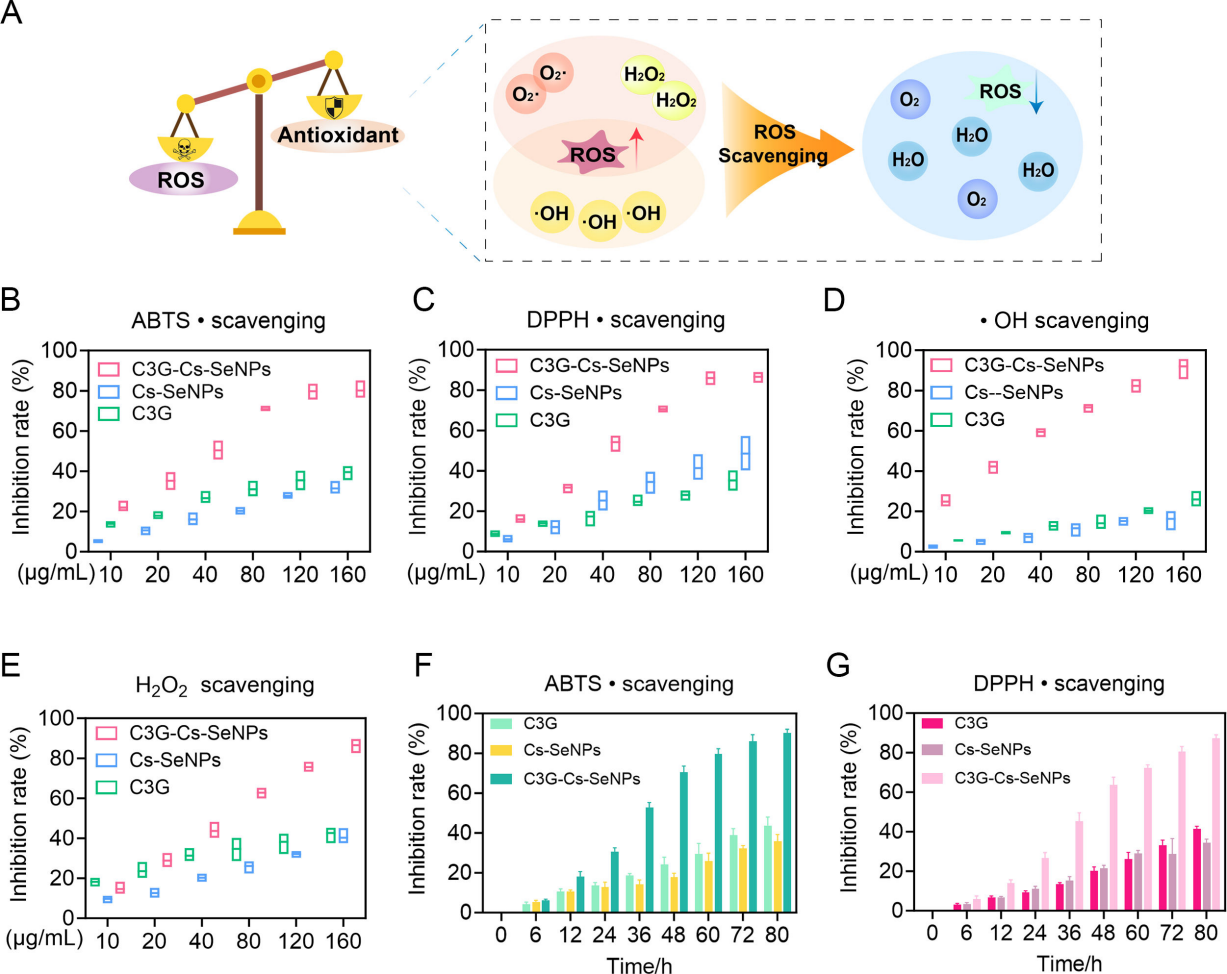
Antioxidant performances of C3G-Cs-SeNPs *in vitro*. (**A**) Schematic illustration of the antioxidation activity of C3G-Cs-SeNPs *in vitro*. (**B through E**) Comparison of the scavenging capacities of C3G, Cs-SeNPs, and C3G-Cs-SeNPs toward ABTS•+ (**B**), DPPH• (**C**), •OH (**D**), and H_2_O_2_ (**E**) at different concentrations (*n* = 3). (**F and G**) Comparison of the stability of scavenging capacities of C3G, Cs-SeNPs, and C3G-Cs-SeNPs toward ABTS•+ (**F**) and DPPH• (**G**) (*n* = 3).

UV-Vis spectroscopy was used to monitor the spectra. As shown in Fig. 1F, the UV–Vis spectra of C3G showed a maximum absorbance peak at 295 nm, while Cs-SeNPs and C3G-Cs-SeNPs showed a shift in wavelength at 267 and 200 nm, respectively. This result is consistent with previously reported research on UV–visible spectroscopy of Cs-SeNPs showing characteristic peaks at ~281 and ~346 nm (35). In addition, the UV-Vis spectrum of pure C3G (dissolved in deionized water, pH ~7.0) in Fig. 1F primarily shows a peak at 295 nm, corresponding to the π→π* transitions of the aromatic rings in the anthocyanidin structure. Since the UV-Vis analysis was conducted in an aqueous solution (pH ~7.0) to match the conditions for nanoparticle characterization, the absence of the 520 nm peak aligns with the expected behavior of C3G at neutral pH. XRD pattern indicated the formation of amorphous C3G, Cs-SeNPs, and C3G-Cs-SeNPs (Fig. 1G). The elemental composition and chemical states of Cs-SeNPs and C3G-Cs-SeNPs were analyzed by XPS, confirming the presence of carbon (C), oxygen (O), and selenium (Se) (Fig. 1H) corresponding to the binding energy of C1s (Fig. 1I and J), O1s (Fig. 1K and L) and Se 3d (Fig. 1M and N), respectively. Strong peaks were observed at 285.98 (Fig. 1I), 530.88 (Fig. 1K), and 53.48 eV (Fig. 1M) in the XPS spectra, providing typical evidence that the Cs-SeNPs consisted of C, O, and Se components, respectively, consistent with previous research (21). Similar peaks were also observed at 284.88 (Fig. 1J), 531.88 (Fig. 1L), and 57.08 eV (Fig. 1N) in C3G-Cs-SeNPs. High-resolution C1s spectra further identified the peaks at 282.8, 285.98, and 286.18 eV, along with 283.28, 284.88, and 286.28 eV, assigning, respectively, to C–C, C = C, and C–O bonds (Fig. 1I and J) as previously reported (36). Finally, the investigation into the particle size stability of Cs-SeNPs and C3G-Cs-SeNPs across various solutions showed that H_2_O and 0.9% NaCl, PBS, and DMEM provide stable conditions with no significant size change (Fig. 1O and P). The enhanced stability of C3G-Cs-SeNPs in physiological media contrasts with the aggregation observed in unmodified SeNPs, likely due to chitosan’s cationic nature and C3G’s ability to form hydrogen bonds, which stabilize the nanoparticle surface. This stability translates to prolonged antioxidant activity, as evidenced by sustained ROS scavenging over 48 hours (Fig. 2F and G), a feature not reported for PEG- or ZnO-based SeNPs (25–27).

### ROS scavenging activity of C3G-Cs-SeNPs

PRRSV infection in Marc-145 cells leads to oxidative stress characterized by the generation of ROS. It has recently been reported that SeNPs can suppress PRRSV-induced apoptosis in Marc-145 cells via the ROS/JNK signaling pathway (21). Consistent with this finding, we measured the ROS scavenging activity of C3G-Cs-SeNPs using ABTS•+, •OH, and H_2_O_2_ and further assessed the antioxidant properties of C3G-Cs-SeNPs using DPPH• (Fig. 2A). The results showed that C3G-Cs-SeNPs significantly wiped out ABTS•+ at a concentration of 120 µg/ mL (Fig. 2B) and DPPH• at a concentration of 80 µg/mL (Fig. 2C). A similar investigation has been done with H_2_O_2_ and •OH radicals, and the result showed that C3G-Cs-SeNPs wiped out 80% of H_2_O_2_ at 120 µg/mL and 73% of •OH at a concentration of 80 µg/mL (Fig. 2D and E), respectively, suggesting its potential catalase-like (CAT-like) and superoxide dismutase-like (SOD-like) activity. Interestingly, by investigating the antioxidant properties of Cs-SeNPs and C3G alone in wiping out ABTS•+ and DPPH• radicals, we found that C3G-Cs-SeNPs showed higher antioxidant activity (Fig. 2F and G), which increased over time compared to C3G and Cs-SeNPs. Similarly, r-PRRSV-EGFP has been reported to increase the production of ROS and H_2_O_2_ and reduce GSH generation (21). In addition, it further showed that PRRSV infection increased ROS levels in Marc-145 cells, whereas treatment with ZnO-Se NPs significantly reduced PRRSV-induced ROS levels in a dose-dependent manner (27). Taken together, we demonstrated that C3G-Cs-SeNPs can suppress the production of ROS, indicating their potential antioxidant properties.

### C3G-Cs-SeNPs impair PRRSV proliferation in Marc-145 cells

To investigate the antiviral activity of C3G-Cs-SeNPs against PRRSV proliferation and define the experimental conditions, we assessed the biocompatibility of C3G, Cs-SeNPs, and C3G-Cs-SeNPs in Marc-145 cells and rPAMs using the CCK-8 assay. The results revealed that all tested substances exhibited excellent biocompatibility, showing minimal cytotoxic effects on both the Marc-145 cells (Fig. S1Athrough C) and rPAMs (Fig. S1D through F). As reported in previous research, nanocarriers, including Cs-SeNPs, utilized for delivering C3G, showed safety, biocompatibility, and high stability of C3G in acidic and high-temperature conditions (17, 37). Similarly, ZnO-SeNPs, although effective against PRRSV, showed notable cytotoxicity to Marc-145 cells at 5 µg/mL after 48 hours, with viability below 80% (27). By contrast, C3G-Cs-SeNPs maintained over 90% viability at the same concentration without apparent cytotoxicity (Fig. S1).

**Fig 3:**
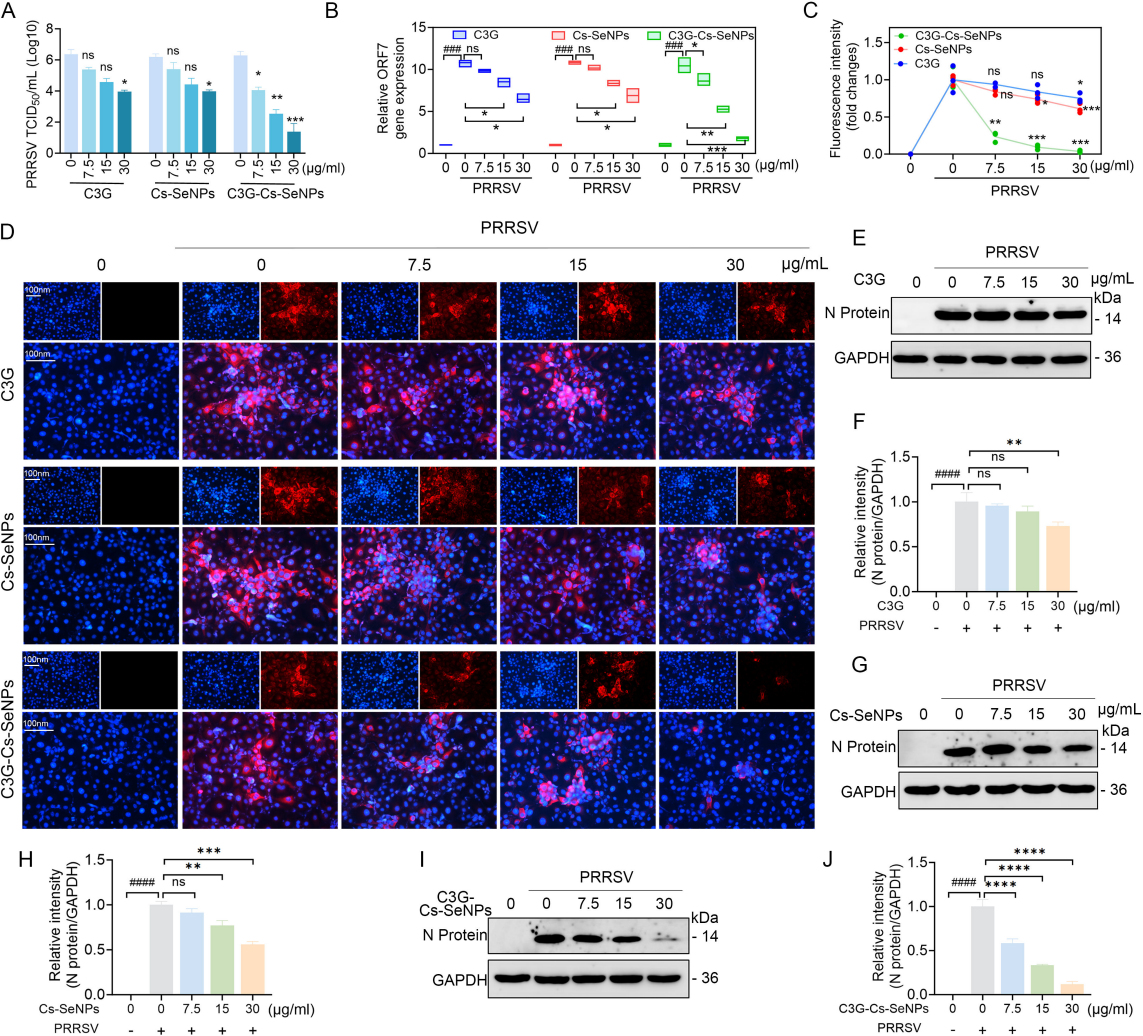
Antiviral activity of C3G, Cs-SeNPs, and C3G-Cs-SeNPs on PRRSV. Marc-145 cells were infected with PRRSV at an MOI of 0.1 for 1 hour, followed by the treatments with C3G, Cs-SeNPs, and C3G-Cs-SeNPs for 48 h. (**A**) The TCID_50_ assay showed PRRSV titers after different treatment concentrations. (**B**) RT-qPCR showed mRNA expression levels of ORF7 after different treatment concentrations. (**C and D**) IFA showed the fluorescence intensity of PRRSV-infected Marc-145 cells after different treatment concentrations. The nucleus was stained with DAPI (blue), and PRRSV N protein was labeled with the fluorescent dye Alexa Fluor 594 (red). Scale bars = 100 µm. Blue represents the nucleus, and red represents the N protein of PRRSV. Scale bar = 100 µm. (**E through J**) Western blot analysis and quantification of protein bands of the PRRSV N protein expression levels after different treatment concentrations of C3G (**E and F**), Cs-SeNPs (**G and H**), and C3G-Cs-SeNPs (**I and J**). All results are means ± SD from three independent experiments performed in triplicate. *P < 0.05, **P < 0.01, ***P < 0.001 vs. indicated group.

Furthermore, based on the above cytotoxicity test results, we investigated the effect of C3G, Cs-SeNPs, and C3G-Cs-SeNPs on PRRSV proliferation. Marc-145 cells were infected with PRRSV at an MOI of 0.1 for 1 hour, followed by the treatments with C3G, Cs-SeNPs, and C3G-Cs-SeNPs, respectively, for 48 h. At the end of the incubation period, the cells were collected and used for TCID_50_, WB, RT-qPCR, and IFA analysis. The C3G and Cs-SeNPs treatment showed relatively stable TCID_50_ levels at 7.5 and 15 µg/mL, compared with the control group, which only reduced the TCID_50_ levels of PRRSV at 30 µg/mL (Fig. 3A). However, C3G-Cs-SeNPs increased effectiveness in reducing PRRSV TCID_50_ levels in a dose-dependent way. RT-qPCR results showed that C3G-Cs-SeNPs significantly reduced the ORF7 mRNA expression levels of PRRSV dose-dependently. Similarly, C3G and Cs-SeNPs treatments only have inhibitory effects at high concentrations of 15 and 30 µg/mL (Fig. 3B). While PEG-SeNPs require higher concentrations of 16 µM to suppress viral replication (25), C3G-Cs-SeNPs achieve significant PRRSV inhibition at 15 µg/mL (Fig. 3A and B).

Meanwhile, IFA showed that C3G-Cs-SeNPs could dose-dependently reduce the N protein fluorescence signal of PRRSV, while C3G and Cs-SeNPs only had inhibitory effects at high concentrations of 30 µg/mL (Fig. 3C and D). These results are consistent with those observed in WB, where the N protein expression levels of PRRSV exhibited inhibitory effects at high concentrations of 30 µg/mL for C3G (Fig. 3E and F) and Cs SeNPs (Fig. 3G and H). However, this expression was significantly reduced by C3G-Cs-SeNPs in a dose-dependent manner (Fig. 3I and J). Consistently, the treatment with CS-SeNPs has been reported to reduce the RNA levels of r-PRRSV-EGFP (21). Furthermore, research has shown that ZnO-SeNPs inhibit PRRSV proliferation, suggesting that the self-assembly ZnO-based SeNPs possess antiviral activity against PRRSV (27). Taken together, this study demonstrated that the self-assembly-based C3G-Cs-SeNPs exhibited a significant inhibitory effect on PRRSV in Marc-145, suggesting the therapeutic potential of C3G-Cs-SeNPs in viral infection.

To comprehensively assess the wide-ranging antiviral activity of C3G-Cs-SeNPs, we tested their antiviral efficacy against two genetically distinct PRRSV strains, PRRSV-1 (BB0907) and PRRSV-2 (S1), in addition to the previously investigated PRRSV-2 strain FJ1402. Result revealed significant suppression of PRRSV N protein expression in cells treated with C3G-Cs-SeNPs during infection with both PRRSV-1 and PRRSV-2 variants (Fig. S2A, D and G). Quantification of PRRSV N protein expression levels (Fig. S2B, E and H) demonstrated a dose-dependent decrease in all strains, with statistical significance compared to untreated controls. Consistently, RT-qPCR results confirm that C3G-Cs-SeNPs also significantly decreased viral replication, as evidenced by reduced transcription of the ORF7 gene in cells infected with BB0907, S1, and FJ1402 strains (Fig. S2C, F and I). These findings collectively confirm the ability of C3G-Cs-SeNPs to inhibit various PRRSV strains, emphasizing their potential as a flexible therapeutic strategy for reducing infections caused by genetically diverse PRRSV isolates.

### C3G-Cs-SeNPs inhibit PRRSV invasion in a multi-stage manner in Marc-145 cells

**Fig 4:**
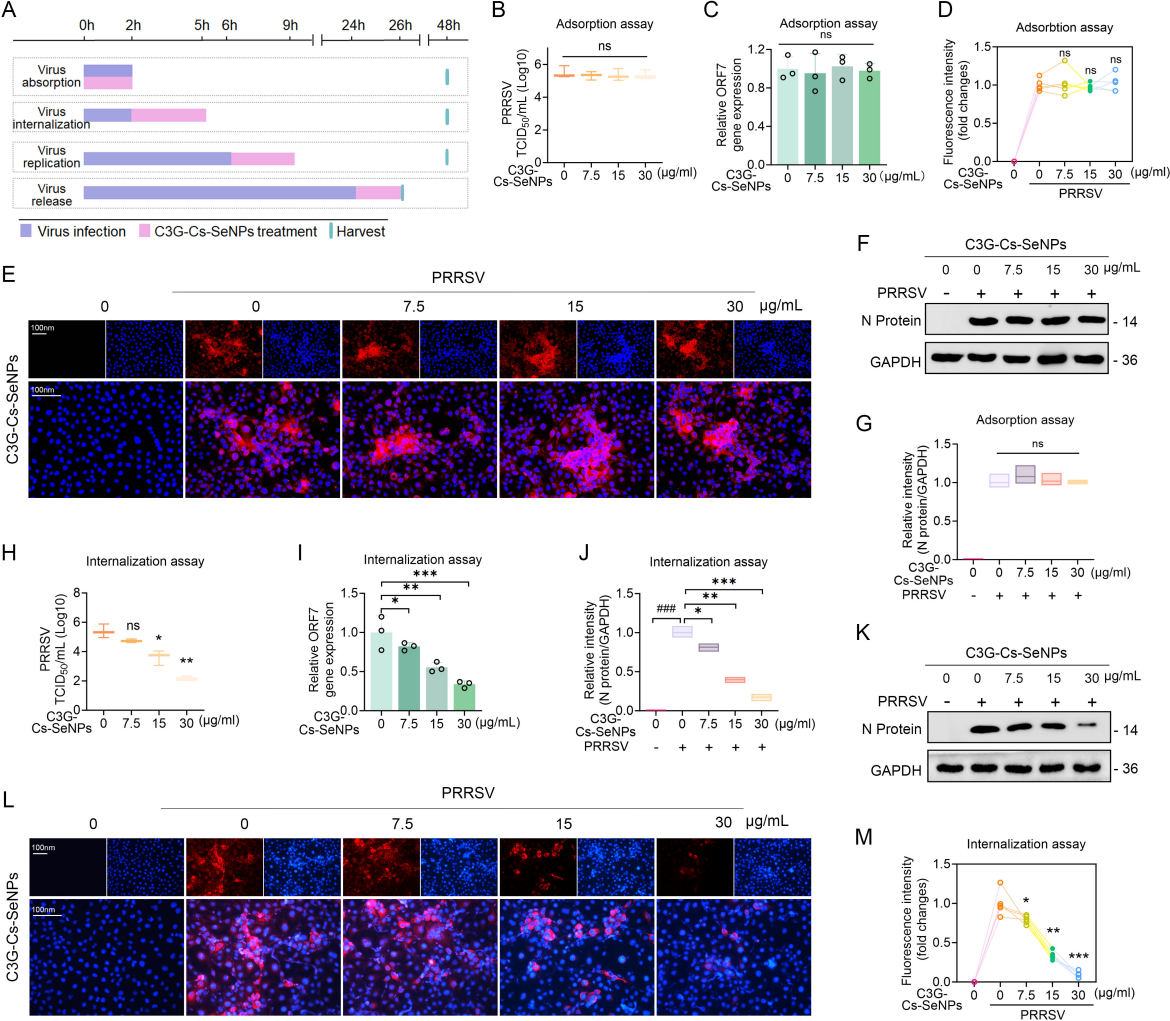
C3G-Cs-SeNPs inhibit PRRSV internalization. (**A**) A schematic diagram illustrates the stages of PRRSV infection alongside C3G-Cs-SeNPs treatment. (**B**) The TCID_50_ assay showed PRRSV titers after different concentrations of C3G-Cs-SeNPs in the adsorption phase. (**C**) RT-qPCR showed mRNA expression levels of ORF7 after different concentrations of C3G-Cs-SeNPs. (**D and E**) IFA showed the fluorescence intensity of PRRSV-infected Marc-145 cells after different concentrations of C3G-Cs-SeNPs. (**F and G**) Western blot analysis showed the expression levels of N proteins after different concentrations of C3G-Cs-SeNPs in the adsorption phase. (**H**) The TCID_50_ assay showed PRRSV titers after different concentrations of C3G-Cs-SeNPs in the internalization phase. (**I**) RT-qPCR showed mRNA expression levels of ORF7 after different concentrations of C3G-Cs-SeNPs in the internalization phase. (**J and K**) Western blot analysis showed the expression levels of N proteins after different concentrations of C3G-Cs-SeNPs. (**L and M**) IFA showed the fluorescence intensity of PRRSV-infected Marc-145 cells after different concentrations of C3G-Cs-SeNPs. The nucleus was stained with DAPI (blue), and PRRSV N protein was labeled with the fluorescent dye Alexa Fluor 594 (red). Scale bar = 100 µm. Means ± SD from three independent experiments performed in triplicate. *P < 0.05, **P < 0.01, ***P < 0.001 vs. indicated group.

PRRSV-infected hosts undergo stages that involve the binding, internalization, uncoating, replication, assembly, and release of the PRRSV virion (38, 39). To investigate the mechanisms by which C3G-Cs-SeNPs inhibit PRRSV invasion in Marc-145 cells, we assessed the effects of C3G-Cs-SeNPs on specific stages of viral infection (Fig. 4A).

Marc-145 cells were exposed to PRRSV at 4°C in the presence or absence of C3G-Cs-SeNPs. Since this temperature allows viral binding without internalization, C3G-Cs-SeNPs did not significantly impair the ORF7 mRNA expression levels at a concentration ranging from 7.5 to 30 µg/mL, evidenced by TCID_50_ values (Fig. 4B and C). The IFA also aligned with these results, showing a heightened viral fluorescence signal of PRRSV (Fig. 4D and E). Furthermore, WB results are consistent with the above findings, showing no significant difference in the N protein expression after different concentrations of C3G-Cs-SeNPs treatment (Fig. 4F and G). The lack of significant inhibition in virus replication suggests that C3G-Cs-SeNPs do not interfere with the adsorption phase of the PRRSV infection (Fig. 4A). Consistently, PRRSV binds to host cell receptors like CD163 or heparan sulfate upon adsorption but does not yet enter the cell (38). This suggests that C3G-Cs-SeNPs do not interfere with the structural interaction between viral surface proteins and host receptors. Unlike polyphenols that directly block receptor-binding domains like SARS-CoV-2 spike protein inhibitors (40), C3G-Cs-SeNPs likely exert their effects downstream of initial attachment. However, in the internalization phase, C3G-Cs-SeNPs significantly reduced the ORF7 mRNA expression levels at a concentration ranging from 7.5 to 30 µg/mL, leading to a significant decrease in PRRSV titer, evidenced by lower TCID_50_ values (Fig. 4A, H and I). WB results further supported these findings, revealing decreased levels of the N protein in a dose-dependent manner at 2 hours of treatment with C3G-Cs-SeNPs ranging from 7.5 to 30 µg/mL (Fig. 4J and K). The IFA also aligned with these results, showing a decrease in the N protein fluorescence signal of PRRSV (Fig. 4L and M). This dose-dependent effect indicates that C3G-Cs-SeNPs likely play a role in inhibiting the internalization of PRRSV into Marc-145 cells, thereby preventing viral entry and subsequent replication. After adsorption, PRRSV enters cells via clathrin-mediated endocytosis or other endocytic pathways (38). C3G-Cs-SeNPs, significantly inhibiting the internalization of PRRSV, may arise first from the modulation of host cell pathways. C3G-Cs-SeNPs activate the SIRT1/Nrf2 pathway (Fig. 8), which regulates redox homeostasis and cellular stress responses. Enhanced antioxidant signaling via HO-1, SOD could destabilize the endosomal environment required for viral uncoating or disrupt ROS-dependent signaling critical for endocytosis. Second, the nanoparticles may alter membrane dynamics or inhibit key proteins like clathrin-dependent endocytosis (CME) involved in internalization (38). Similar mechanisms have been reported for selenium nanoparticles, which impair viral entry by modulating lipid rafts or cytoskeletal rearrangements (41). Targeting the binding of the virus to the host receptor is an effective strategy to prevent viral entry and subsequent infection. Evidence has shown that polyphenols, specifically phenolic compounds, displayed the highest binding with the receptor-binding domain of spike protein, inhibiting viral attachment to the human angiotensin-converting enzyme two receptors and, thus, cellular entry of pseudo-typed SARS-CoV-2 virions (40). Overall, this study demonstrated that polyphenol-based nanoparticles like C3G-Cs-SeNPs do not competitively inhibit viral attachment but may alter cellular mechanisms affecting virus entry. This suggests potential interference with receptor-mediated endocytosis or disruption of signaling pathways involved in viral uptake.

**Fig 5:**
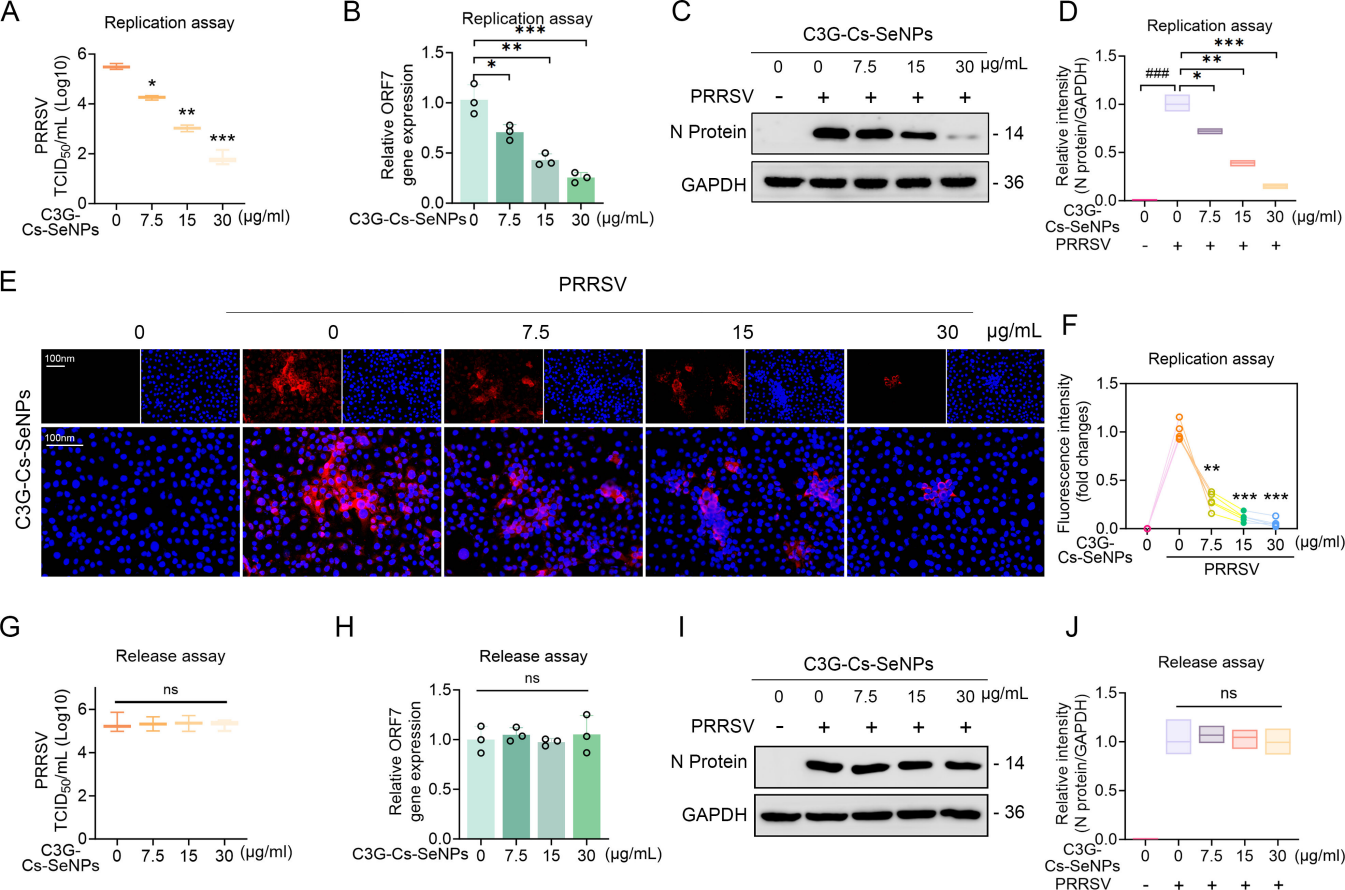
C3G-Cs-SeNPs inhibit PRRSV replication. (**A**) The TCID_50_ assay showed PRRSV titers after different concentrations of C3G-Cs-SeNPs in the replication phase. (**B**) RT-qPCR showed mRNA expression levels of ORF7 after different concentrations of C3G-Cs-SeNPs. (**C and D**) Western blot analysis showed the expression levels of N proteins after different concentrations of C3G-Cs-SeNPs. (**E and F**) IFA showed the fluorescence intensity of PRRSV-infected Marc-145 cells after different concentrations of C3G-Cs-SeNPs in the replication phase. The nucleus was stained with DAPI (blue), and PRRSV N protein was labeled with the fluorescent dye Alexa Fluor 594 (red). Scale bar = 100 µm. C3G-Cs-SeNPs do not influence PRRSV release. (**G**) The TCID_50_ assay showed PRRSV titers after different concentrations of C3G-Cs-SeNPs in the release phase. (**H**) RT-qPCR showed mRNA expression levels of ORF7 after different concentrations of C3G-Cs-SeNPs. (**I and J**) Western blot analysis of the expression levels of PRRSV N proteins under the treatment of C3G-Cs-SeNPs in the release phase. Means ± SD from three independent experiments performed in triplicate. *P < 0.05, **P < 0.01, ***P < 0.001 vs. indicated group.

Moreover, to explore whether C3G-Cs-SeNPs directly inhibit the replication of viral RNA, PRRSV-infected Marc-145 cells were incubated for 6 hours at 37 °C and then cultured in a fresh medium containing C3G-Cs-SeNPs (Fig. 4A). Cells were collected at 9 hours and used for RT-qPCR analysis. The result showed that C3G-Cs-SeNPs treatment significantly reduced the expression levels of virus RNA, as evidenced by decreased TCID_50_ values (Fig. 5A) and lower relative ORF7 gene expression (Fig. 5B) at concentrations ranging from 7.5 to 30 µg/mL. WB results further confirmed these findings, showing reduced levels of the viral N protein in cells treated with C3G-Cs-SeNPs (Fig. 5C). The quantification of N protein showed a decrease with increasing concentrations of the treatment (Fig. 5D), demonstrating a direct correlation between the concentration of C3G-Cs-SeNPs and the suppression of viral protein expression. IFA further aligned with these results, showing a decrease in PRRSV N protein fluorescence signal (Fig. 5E and F). Mechanistically, this result implies that Cs-SeNPs incorporated into C3G-Cs-SeNPs may directly interfere with PRRSV replication by disrupting viral RNA synthesis and structural protein assembly.

To investigate whether C3G-Cs-SeNPs could impair the release of PRRSV, Marc-145 cells were infected with PRRSV for 24 hours at 37 °C, followed by 2 hour of culture in fresh medium containing different concentrations of C3G-Cs-SeNPs (Fig. 4A). The result showed that C3G-Cs-SeNPs did not significantly affect PRRSV release from infected cells, as evidenced by the equivalent TCID_50_ values (Fig. 5G) and unchanged ORF7 expression levels (Fig. 5H) across the tested concentrations. In addition, WB results demonstrated no significant inhibition in the levels of N protein (Fig. 5I and J). PRRSV release involves budding from host membranes, a process dependent on viral structural proteins like GP5 and M and host secretory pathways (38). Once viral replication and assembly are complete, the C3G-Cs-SeNPs may not target late-stage processes such as virion packaging or exocytosis. Specifically, the SIRT1/Nrf2 pathway primarily mitigates oxidative stress and inflammation, which are more critical during early infection stages (internalization/replication) than during release. Collectively, C3G-Cs-SeNPs selectively inhibit PRRSV internalization by targeting host-cell mechanisms essential for viral entry (e.g., endocytosis, redox balance) rather than directly blocking viral adsorption or release. This stage-specific activity aligns with their role as host-directed antivirals, emphasizing their potential to disrupt early infection events while sparing later stages that depend on distinct molecular processes.

### C3G-Cs-SeNPs inhibit inflammation in PRRSV-infected Marc-145 cells and rPAMs

**Fig 6:**
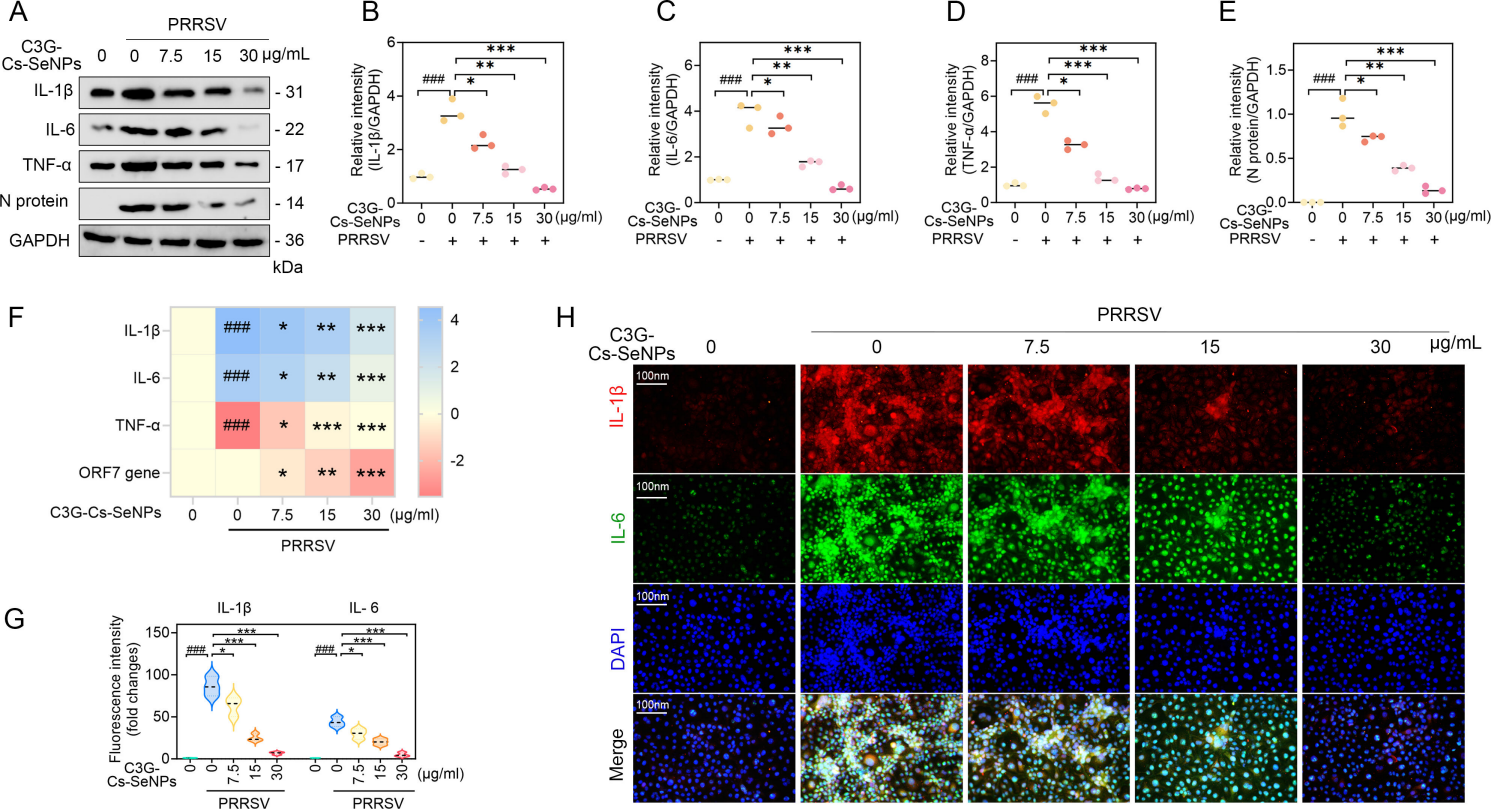
C3G-Cs-SeNPs impair inflammation in Marc-145 cells. Marc-145 cells were treated with PRRSV for 1 hour, followed by C3G-Cs-SeNPs at different concentrations for 24 hours. (**A through E**) Western blot analysis showed the effect of C3G-Cs-SeNPs on the expression levels of inflammatory cytokines in PRRSV-infected Marc-145 cells. (**F**) RT-qPCR showed the effect of C3G-Cs-SeNPs on mRNA expression of inflammatory factors in PRRSV-infected Marc-145 cells. (**G and H**) IFA showed the effects of C3G-Cs-SeNPs on the expression of IL-1β and IL-6 in PRRSV-infected Marc-145 cells. The nucleus was stained with DAPI (blue), and PRRSV N protein was labeled with the fluorescent dye Alexa Fluor 594 (red). Scale bar = 100 µm. *^###^P <* 0.001*; ^##^P <* 0.01*; ^#^P <* 0.05 vs DMSO-treated cells. All results are means ± SD from three independent experiments performed in triplicate. ***P < 0.001; **P < 0.01; *P < 0.05 vs. PRRSV-infected cell groups.

PRRSV infection triggers the release of various pro-inflammatory factors, significantly contributing to the pathogenesis and inflammatory responses. Studies have shown that PRRSV infection increases levels of key cytokines, such as IL-1β, TNF-α, and IL-6, indicating an upregulation of the inflammatory response in infected cells (42–44). This modulation occurs particularly in the early stages of infection, affecting the immune response. To investigate this, we hypothesized that C3G-Cs-SeNPs may affect the expression of pro-inflammatory factors in PRRSV-infected cells. To confirm the results from Marc-145 cells, we investigated rPAMs and yielded similar results. As an immortalized porcine alveolar macrophage line, rPAMs provide consistent results by overcoming primary PAM limitations like donor variability and limited viability. They retain essential macrophage functions, including PRRSV susceptibility, making them a reliable model for studying viral pathogenesis and therapies (45, 46). The WB results showed that C3G-Cs-SeNPs treatment, at concentrations ranging from 7.5 to 30 µg/mL, significantly reduced the protein expression levels of IL-1β, IL-6, and TNF-α, which are typically upregulated during viral infections (Fig. 6A through E and Fig. S3A through E). RT-qPCR aligned with WB results, showing decreased mRNA expression levels of IL-1β, IL-6, and TNF-α (Fig. 6F and Fig. S3Fthrough I). This downregulation indicates that C3G-Cs-SeNPs may play a crucial role in modulating the inflammatory responses induced by PRRSV infection. Meanwhile, the IFA results are consistent with WB and RT-qPCR results, showing a significant decrease in the immunofluorescence signal of IL-1β and IL-6 in Marc-145 cells after treatment with C3G-Cs-SeNPs at concentrations ranging from 7.5 to 30 µg/mL (Fig. 6G and H).

More significantly, it has been reported that SeNPs can activate the immune system, which results in an immunological response that fights various diseases caused by chronic inflammation (47). Recently, research has shown that polyethylene glycol (PEG)-modified gray selenium nanoparticles (PEG-SeNPs) inhibited the production of pro-inflammatory factors such as IL-1β, IL-5, IL-6, and TNF-α in the cells infected with influenza A virus (H1N1) (25). In addition, the self-assembly of FA-OSAS-SeNPs has been reported to significantly suppress the release of inflammatory factors such as IL-6, IL-12, IL-1β, and TNF-α (35). Collectively, our findings demonstrated that C3G-Cs-SeNPs possess anti-inflammatory properties, potentially mitigating the severe inflammatory response often triggered by viral infections. Elevated levels of IL-1β, IL-6, and TNF-α are commonly associated with the pathogenesis of viral diseases, contributing to tissue damage and exacerbating the clinical manifestations of infection. The effective reduction of these cytokines could, thus, promote a more favorable outcome in viral infection. Moreover, these results suggest that C3G-Cs-SeNPs could serve as a potential therapeutic strategy for managing PRRSV-induced inflammation.

### C3G-Cs-SeNPs inhibit ferroptosis and oxidative stress in PRRSV-infected cells

**Fig 7:**
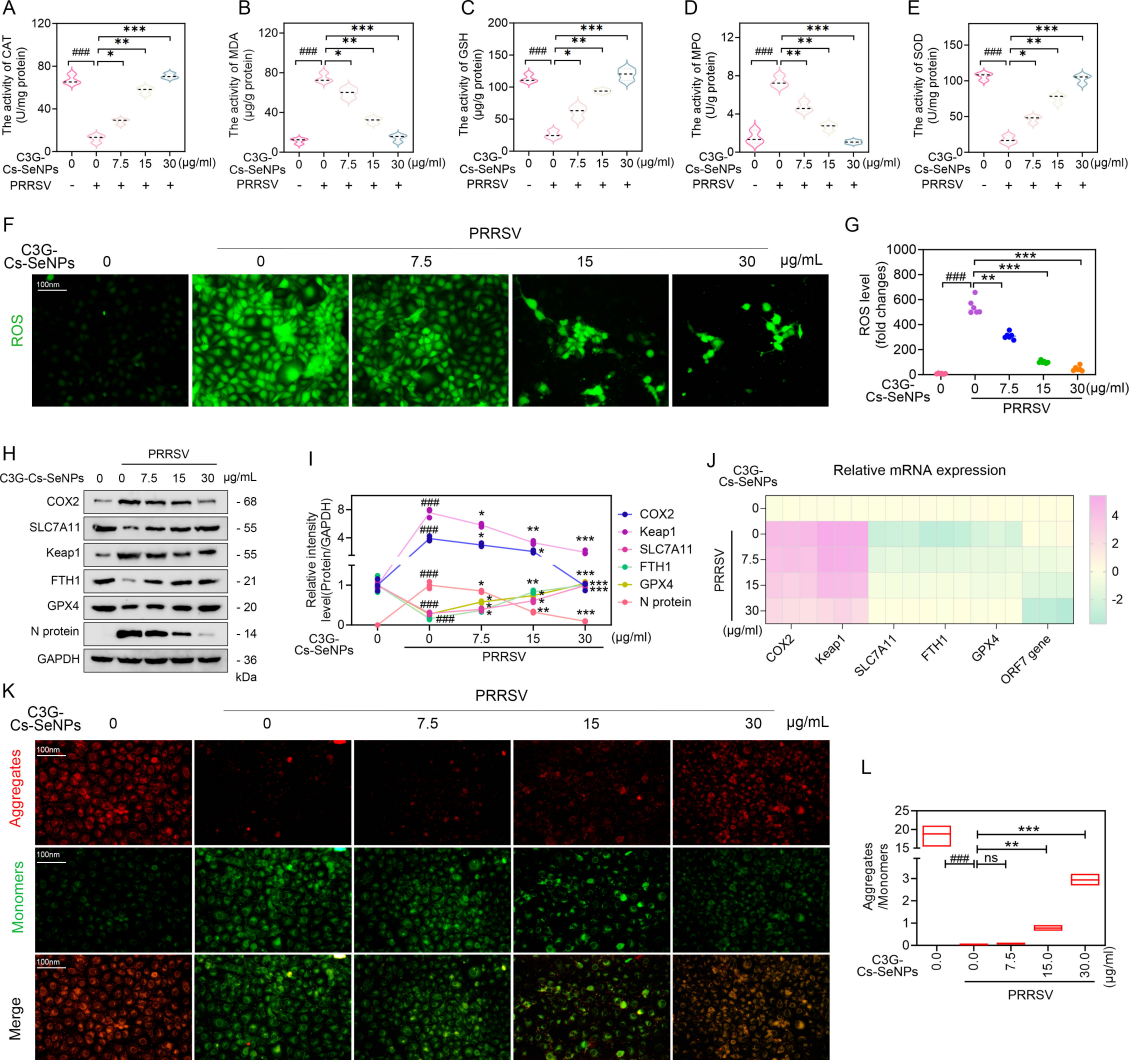
C3G-Cs-SeNPs inhibit ferroptosis in PRRSV-infected Marc-145 cells. (**Athrough E**) Effects of C3G-Cs-SeNPs on CAT (**A**), MDA (**B**), GSH (**C**), MPO (**D**), and SOD (**E**) levels in PRRSV-infected Marc-145 cells. (**F and G**) Effects of C3G-Cs-SeNPs on ROS levels in Marc-145 cells infected by PRRSV. (**H and I**) Western blot showed the effect of C3G-Cs-SeNPs on the protein expression of ferroptosis-related markers. (**J**) RT-qPCR showed the mRNA expression of COX2, SLC7A11, Keap1, FTH1, GPX4, and ORF7 gene in PRRSV-infected Marc-145 cells. (**K and L**) IFA showed the effects of C3G-Cs-SeNPs on the mitochondrial membrane potential. The nucleus was stained with DAPI (blue), and PRRSV N protein was labeled with the fluorescent dye Alexa Fluor 594 (red). Scale bar = 100 µm. All results are means ± SD from three independent experiments performed in triplicate. *^###^P <* 0.001*; ^##^P <* 0.01*; ^#^P <* 0.05 vs DMSO-treated cells. ***P < 0.001; **P < 0.01; *P < 0.05 vs. PRRSV-infected cell groups.

Viral infection induces oxidative stress by generating ROS (48–50), which can subsequently lead to ferroptosis (33, 51). Consistent with these findings, it is crucial to assess the effect of C3G-Cs-SeNPs on oxidative stress in PRRSV-infected cells by measuring oxidative stress-associated biomarkers. As shown in Fig. 7A through E, PRRSV significantly increased MDA and MPO levels while decreasing the activities of SOD, CAT, and GSH in Marc-145 cells, suggesting enhanced oxidative stress. Meanwhile, treatment with C3G-Cs-SeNPs reduced MDA and MPO levels and restored SOD, CAT, and GSH activities in a dose-dependent manner. Notably, treatment with C3G-Cs-SeNPs, at concentrations ranging from 7.5 to 30 µg/mL, significantly enhanced the activity of antioxidant enzymes, suggesting their potential to reduce oxidative damage caused by PRRSV. Typically, the generation of ROS leads to oxidative stress that promotes virus replication (52). While ROS is primarily generated in the mitochondria (53), we assessed ROS levels in Marc-145 cells by fluorescence staining using the ROS-sensitive probe DCFH-DA. PRRSV-infected cells significantly increased ROS generation in the fluorescence signal (Fig. 7F and G). However, treatment with C3G-Cs-SeNPs at concentrations ranging from 7.5 to 30 µg/mL significantly decreased the fluorescence signals of ROS (48). These results align with substantial evidence that PRRSV induces oxidative stress in Marc-145 cells and PAMs (47). Recent research showed that r-PRRSV-EGFP increases the production of ROS and decreases GSH generation, while CS-SeNPs treatment restores GSH levels in infected cells (21). In addition, PEG-SeNPs have been shown to inhibit ROS production following H1N1 infection (25). Oxidative stress is typically defined by elevated levels of specific biomarkers, such as MDA and lipid hydroperoxides, alongside decreased levels of antioxidant defense enzymes, including CAT, SOD, and GPx (33). This finding aligned with this study, demonstrating that C3G-Cs-SeNPs could effectively counter PRRSV-induced ROS generation and retain redox homeostasis.

Iron overload leads to oxidative stress due to an imbalance between the production and elimination of ROS (33). While oxidative stress and ferroptosis are interconnected due to similar pathological processes, as previously reported (8, 9, 54, 55), comprehensive research on their crosstalk in PRRSV infection is lacking. Thus, we investigated the effect of C3G-Cs-SeNPs on the key ferroptosis-related biomarkers in PRRSV-infected cells. As expected, PRRSV significantly led to increased protein expression levels of COX2 and SLC7A11 and decreased levels of Keap1, FTH1, and GPX4 (Fig. 7H and I, and Fig. S4A through G), suggesting PRRSV-induced ferroptosis in both Marc-145 cells and rPAMs. However, treatment with C3G-Cs-SeNPs at 7.5 to 30 µg/mL significantly reduced COX2 and Keap protein levels while restoring SLC7A11, FTH1, and GPX4 levels. These findings suggest that C3G-Cs-SeNPs inhibit ferroptosis in PRRSV-infected Marc-145 cells and rPAMs by regulating redox homeostasis. RT-qPCR results aligned with WB results showing that C3G-Cs-SeNPs significantly decreased the mRNA expression levels of COX2, Keap1, and PRRSV N protein, whereas promoting SLC7A11, FTH1, and GPX4 expression (Fig. 7J and Fig. S4H through M). These findings further demonstrate that Cs-SeNPs derived from C3G-Cs-SeNPs integrate into selenoproteins such as GPX4, enhancing endogenous antioxidant defenses, thus mitigating virus-induced oxidative stress.

Ferroptosis is an iron-dependent mechanism (6, 56), and cells with partial mitochondrial depletion remain sensitive to ferroptosis (57, 58). This suggests that impaired mitochondrial function may affect the generation of iron ions (Fe^2+^). Given the critical role of mitochondria in ROS production and their association with ferroptosis (59), we found great significance in assessing mitochondrial membrane potential changes in Marc-145 cells using JC-1 staining. The results showed that PRRSV significantly depolarized the mitochondrial membrane, suggesting mitochondrial dysfunction. However, treatment with C3G-Cs-SeNPs at concentrations ranging from 7.5 to 30 µg/mL significantly restored the mitochondrial membrane (Fig. 7K and L). These results suggest that C3G-Cs-SeNPs preserve mitochondrial function, supporting their role in inhibiting ferroptosis. Consistently, lipid peroxidation is crucial in the ferroptosis process, with MDA, a byproduct, causing harmful modifications to proteins and nucleic acids that trigger cell death (6, 56, 60).

Excess Fe^2+^ produces ROS through the Fenton reaction and activates iron-containing enzymes like lipoxygenase, further promoting lipid peroxidation and ferroptosis (5). Recent studies have shown that C3G inhibits ferroptosis in ischemia/reperfusion acute kidney injury (I/R-AKI) models, reversing excessive intracellular Fe^2+^ accumulation and ROS production while increasing glutathione peroxidase 4 (GPX4) and GSH levels (14). Furthermore, Cs-SeNPs have shown efficacy in inhibiting ferroptosis and cadmium-induced hepatotoxicity by decreasing intracellular Fe^2+^, lipid peroxidation, and oxidative stress (22). Typically, key features of ferroptosis include impaired lipid peroxide scavenging, excess redox-active Fe^2+^, lipid peroxide accumulation, and mitochondrial damage (61). Overall, this evidence is consistent with the findings of this study, as a key contributor to the mechanism, the synergetic effect of C3G and Cs-SeNPs neutralizes ROS generated during PRRSV infection, reducing lipid peroxidation and mitochondrial damage, thus inhibiting ferroptosis.

### C3G-Cs-SeNPs inhibit inflammation and ferroptosis in PRRSV-infected cells through the SIRT1/Nrf2 signaling pathway

Silent information regulator 2 homolog 1 (SIRT1) is a nicotinamide adenine dinucleotide (NAD^+^)-dependent mitochondrial deacetylase critical for regulating mitochondrial reactive oxygen species (ROS) and oxidative stress through the acetylation of transcription factors (62). It plays a vital role in various cellular functions such as energy metabolism, inflammation, and redox homeostasis, with nuclear factor erythroid 2-related factor 2 (Nrf2) as a key target, responsible for regulating antioxidant responses. Subsequently, in the nucleus, Nrf2 binds to antioxidant response elements (AREs) to activate downstream antioxidant signaling pathways, such as glutathione peroxidase (GPX), heme oxygenase-1 (HO-1), superoxide dismutase (SOD), quinone oxidoreductase 1 (NQO1) (63–65). HO-1 catalyzes heme degradation, producing biliverdin/bilirubin (antioxidants), and carbon monoxide (anti-inflammatory), which collectively reduce oxidative stress and lipid peroxidation, key drivers of ferroptosis (66).

**Fig 8:**
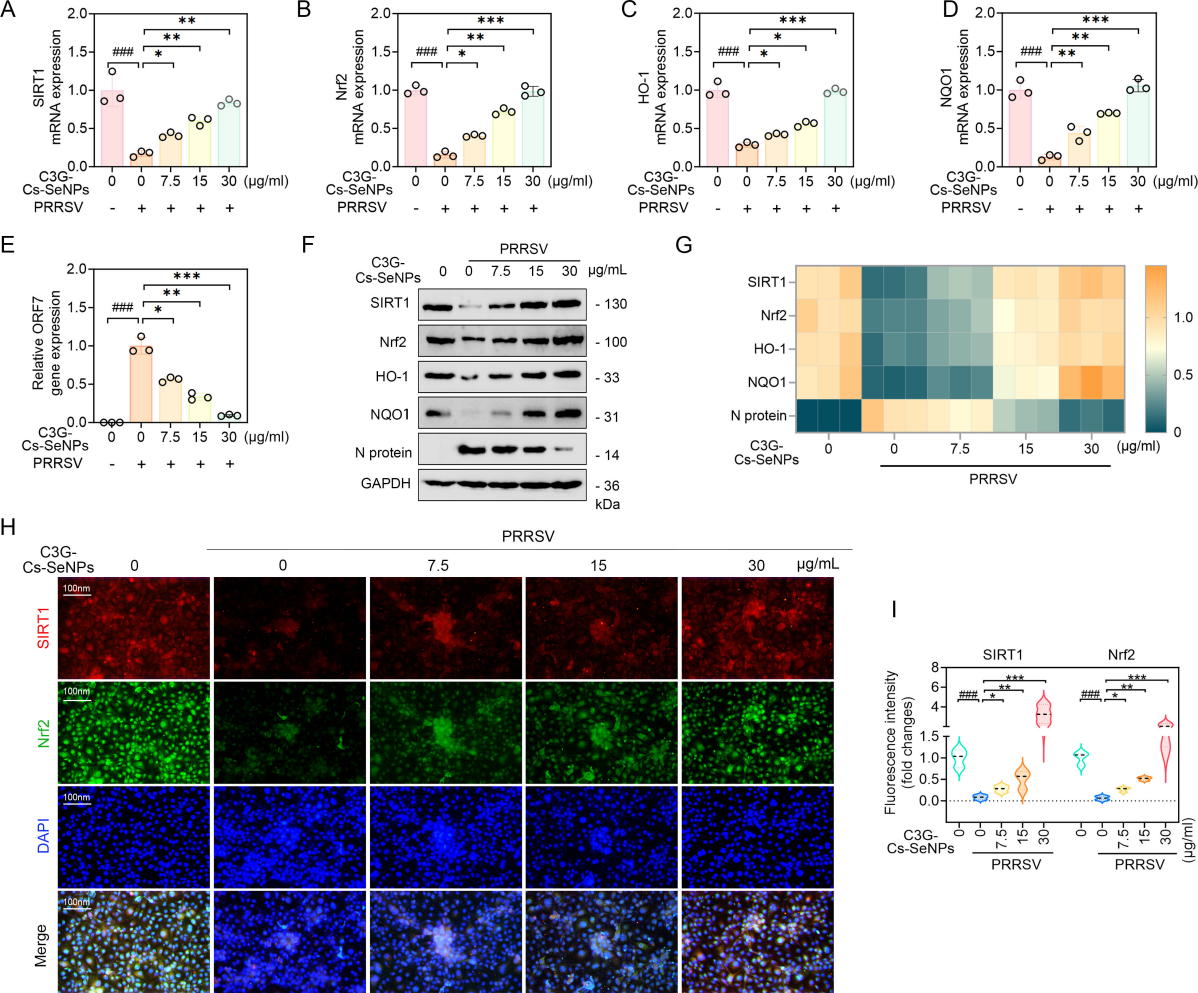
C3G-Cs-SeNPs restore inflammation and ferroptosis in Marc-145 cells infected by PRRSV through the SIRT1/Nrf2 signaling pathway. (**A through E**) RT-qPCR showed the effect of C3G-Cs-SeNPs on mRNA expression of the SIRT1/Nrf2 signaling pathway in PRRSV-infected Marc-145 cells. (**F and G**) Western blot analysis showed the effect of C3G-Cs-SeNPs on the protein expression of the SIRT1/Nrf2 signaling pathway in PRRSV-infected Marc-145 cells. (**H and I**) IFA showed the effects of C3G-Cs-SeNPs on the expression of SIRT1 and Nrf2. The nucleus was stained with DAPI (blue), and PRRSV N protein was labeled with the fluorescent dye Alexa Fluor 594 (red). Scale bar = 100 µm. All results are means ± SD from three independent experiments performed in triplicate. *^###^P <* 0.001*; ^##^P <* 0.01*; ^#^P <* 0.05 vs DMSO-treated cells. ***P < 0.001; **P < 0.01; *P < 0.05 vs. PRRSV-infected cell groups.

SIRT1 signaling plays a significant role in regulating Nrf2 (67, 68), establishing the SIRT1/Nrf2/HO-1 pathway crucial for maintaining redox balance *in vivo,* as evidenced by recent research in some diseases (69–71). However, whether SIRT1/Nrf2 regulates inflammation and ferroptosis in viral infections, particularly respiratory infections like PRRSV, is yet to be elucidated. Thus, we found great importance in investigating whether C3G-Cs-SeNPs could mitigate inflammation and ferroptosis in PRRSV-infected cells via activation of the SIRT1/Nrf2 pathway. As expected, PRRSV infection significantly reduced the mRNA expression levels of SIRT1, Nrf2, HO-1, and NQO1, while treatment with C3G-Cs-SeNPs increased the levels of this mRNA expression and decreased PRRSV N protein (Fig. 8A through E and Fig. S5G through K). WB results aligned with RT-qPCR findings showing that C3G-Cs-SeNPs significantly increased the protein expression levels of SIRT1, Nrf2, HO-1, and NQO1 and decreased PRRSV N protein (Fig. 8F and Fig. S5A). Mechanistically, C3G-Cs-SeNPs enhance Nrf2 stabilization by reducing Keap1-mediated ubiquitination, promoting nuclear translocation of Nrf2. This activates downstream antioxidant genes like HO-1 and NQO1, restoring redox balance.

Furthermore, the quantification of protein intensity also yielded similar results in both PAMs (Fig. S5B) and Marc-145 cells (Fig. 8G). The IFA also aligned with these results, showing an increase in SIRT1 and Nrf2 fluorescence signals after treatment of C3G-Cs-SeNPs (Fig. 8H and I). These results suggest that C3G-Cs-SeNPs enhanced the SIRT1/Nrf2 pathway responses and reduced the extent of cell death caused by iron overload, most likely by regulating genes associated with iron metabolism and the synthesis of antioxidants that protect cells from oxidative stress and ferroptosis. Similarly, it has been shown that the hyperactivation of Nrf2 allows cancer cells to more effectively regulate intracellular iron metabolism, thus inhibiting iron-dependent lipid peroxidation and evading ferroptosis (72). Polyphenol stilbene resveratrol has been shown to increase the expression of SIRT1, Nrf2, and HO-1 while decreasing the expression of rabies virus (RABV) N protein (73). Recently, research revealed that ulinastatin provides hepatoprotection against ferroptosis by activating the SIRT1/Nrf2 signaling pathway (70). Viral infections often lead to increased inflammation and oxidative stress, which cause tissue injury. Current PRRSV treatments, including inactivated vaccines and antiviral drugs like ribavirin, commonly fail due to viral mutation and immune evasion (74, 75). For instance, C3G-Cs-SeNPs reduced viral titers (TCID_50_) by 3.5-log at 30 µg/mL (Fig. 3A), outperforming ribavirin (75), which requires 50 µg/mL (0.2 mM) for comparable effects. Thus, the recent key focus of antiviral research is to develop host-directed medications that reduce inflammation, ferroptosis, ROS accumulation, and viral infectivity. Host-directed antivirals are typically more effective because their targets exist within the stable host genome (63, 76). This study elucidated significant novel mechanisms by which C3G-Cs-SeNPs inhibited inflammation and ferroptosis in PRRSV-infected cells by activating the SIRT1/Nrf2 pathway, suggesting the potential of C3G-Cs-SeNPs as a host-directed antiviral candidate. The synergistic effect of Cs-SeNPs and C3G stems from their complementary mechanisms, with Cs acting as a nanocarrier that improves the stability and cellular uptake of both Se and C3G. The primary mechanism involves SeNPs activating Nrf2 signaling, upregulating antioxidant enzymes such as GPX4 and SOD, while C3G promotes SIRT1-mediated deacetylation of Nrf2. This post-translational modification extends Nrf2’s nuclear retention and enhances its transcriptional activity. Together, these dual actions collectively suppress viral replication, inflammatory responses, and ferroptosis with greater efficacy than either component alone. Although Cs exhibits mild intrinsic antioxidant activity, its principal function lies in optimizing the sustained release and bioavailability of Se and C3G. This controlled delivery mechanism indirectly facilitates the activation of downstream cellular pathways.

More recently, research has provided new insights into crosstalk between the Nrf2 pathway and ferroptosis in lung disease. They reported that the genes involved in the ferroptosis cascade, which are closely linked to the onset of lung diseases, are among the regulatory targets of Nrf2, suggesting potential novel therapeutic mechanisms for lung diseases (77). Overall, this study suggests that C3G-Cs-SeNPs may trigger the SIRT1/Nrf2/HO-1 pathway, helping to regulate inflammation and ferroptosis in PRRSV-infected cells. Elucidating this crosstalk between inflammation, ferroptosis, and SIRT1/Nrf2/HO-1 pathway in the respiratory infection that leads to severe lung injury, like PRRSV, could facilitate the design of new treatments and preventative strategies, such as self-assembled C3G-based SeNPs, for iron-associated illnesses in viral infection.

### SIRT1 knockdown abolishes the protective effects of C3G-Cs-SeNPs by promoting ferroptosis, inflammation, and oxidative stress

**Fig 9:**
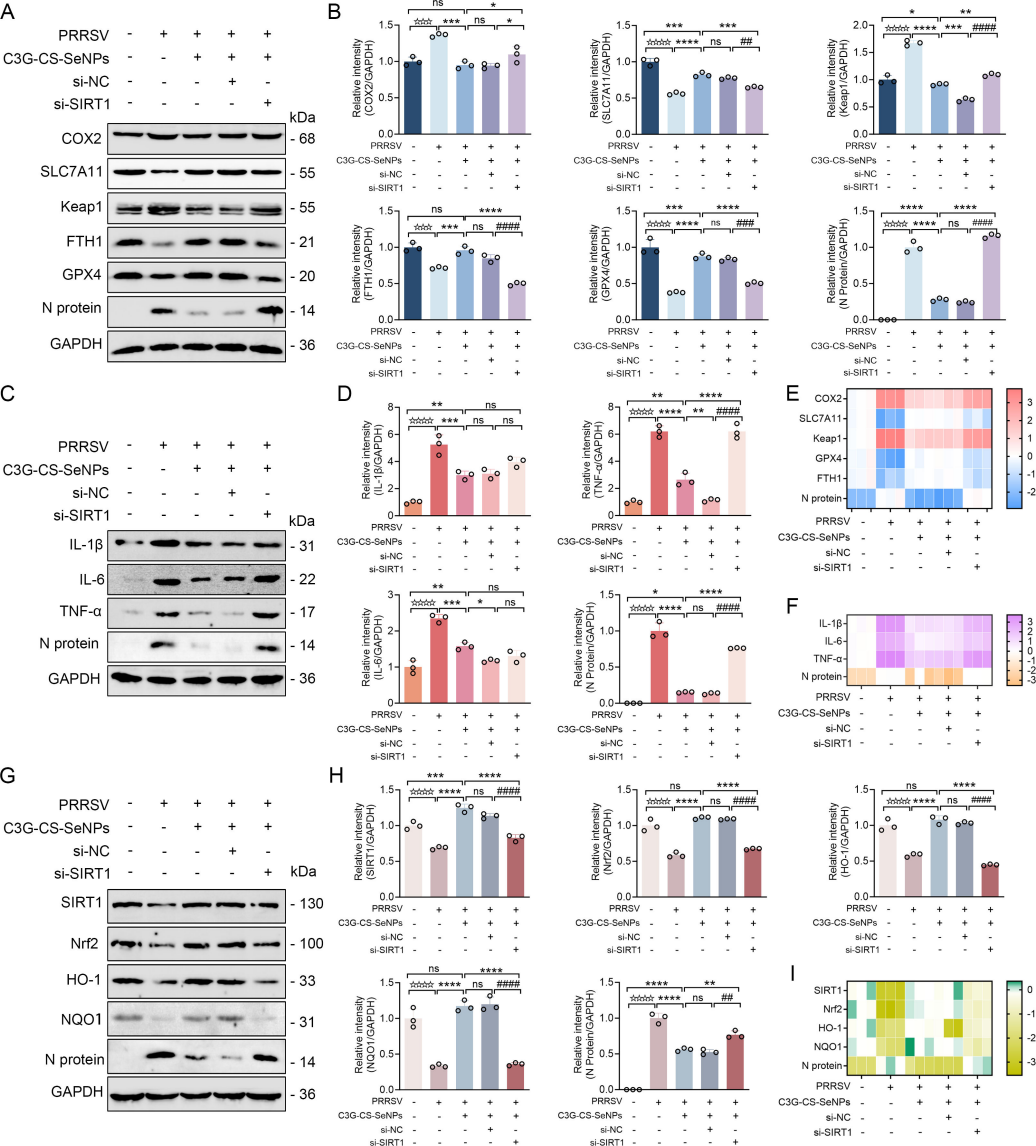
SIRT1 knockdown abolishes the protective effects of C3G-Cs-SeNPs by promoting ferroptosis, inflammation, and oxidative stress. (**A**) Western blot analysis of ferroptosis-related proteins (COX-2, SLC7A11, KEAP1, FTH1, and GPX4) and PRRSV N protein following siRNA-mediated knockdown of SIRT1. (**B**) Quantification of protein bands shown in (**A**) to evaluate relative protein expression. (**C**) Western blot analysis of inflammatory cytokines (IL-1β, IL-6, and TNF-α) after SIRT1 knockdown. (**D**) Quantification of bands shown in (**C**) to evaluate relative protein expression. (**E**) mRNA expression levels of ferroptosis-associated genes (Cox-2, Slc7a11, Keap1, Fth1, and Gpx4) and viral N gene measured by qRT-PCR. (**F**) mRNA expression levels of inflammatory markers (Il-1β, Il-6, and Tnf-α) by qRT-PCR. (**G**) Western blot analysis of key oxidative stress pathway proteins (SIRT1, Nrf2, HO-1, and NQO1) following si-SIRT1-treated cells. (**H**) Quantification of protein bands shown in (**G**). (**I**) mRNA levels of SIRT1, Nrf2, Ho-1, and NQO1 assessed by qRT-PCR. Data are shown as mean ± SD (*n* = 3); *P < 0.05, **P < 0.01, ***P < 0.001 vs. control or indicated groups.

To explore the critical role of SIRT1 in mediating the protective effects of C3G-Cs-SeNPs against PRRSV-infected MARC-145, we conducted siRNA-mediated SIRT1 knockdown and analyzed ferroptosis-related, inflammatory, and redox signaling markers. SIRT1 knockdown significantly upregulated ferroptosis-related proteins COX-2 and Keap1, while decreasing GPX4, SLC7A11, and FTH1 expression (Fig. 9A). Concurrently, PRRSV N protein expression rose significantly, suggesting heightened viral proliferation. Quantification of these makers’ protein expression further confirms this trend with statistical significance (*P* < 0.05; Fig. 9B).

WB results (Fig. 9C) revealed that SIRT1 depletion enhanced the expression of pro-inflammatory cytokines (IL-1β, IL-6, and TNF-α), as evidenced by protein quantification analysis (Fig. 9D). SIRT1 is known to regulate inflammation and stress response, as evidenced by suppression of NF-κB-mediated pro-inflammatory cytokine IL-1β, IL-6, and TNF-α production (78). Similarly, RT-qPCR analyses confirmed these trends, showing elevated transcription of ferroptosis-related mRNA expression (COX-2 and Keap1) and suppressed mRNA expression levels of GPX4 and SLC7A11 (Fig. 9E), alongside upregulated PRRSV N protein and inflammatory cytokine transcripts (Il-1β, IL-6, Tnf-α; Fig. 9F). Notably, SIRT1 silencing attenuated the expression of oxidative stress regulators (Nrf2, HO-1, and NQO1) without affecting the efficiency of SIRT1 knockdown itself (Fig. 9G and H). RT-qPCR analysis confirmed decreased mRNA expression levels of Nrf2, Ho-1, and NQO1 (Fig. 9I).

These findings collectively highlight the pivotal role of SIRT1 in mediating ferroptosis suppression, inflammation resolution, and antioxidant defense during C3G-Cs-SeNPs treatment. The reversal of protective effects upon SIRT1 inhibition underscores its central regulatory function in mitigating viral replication and oxidative damage in PRRSV-infected cells.

### Nrf2 inhibition reverses the protective effects of C3G-Cs-SeNPs by aggravating ferroptosis, inflammation, and oxidative stress

**Fig 10:**
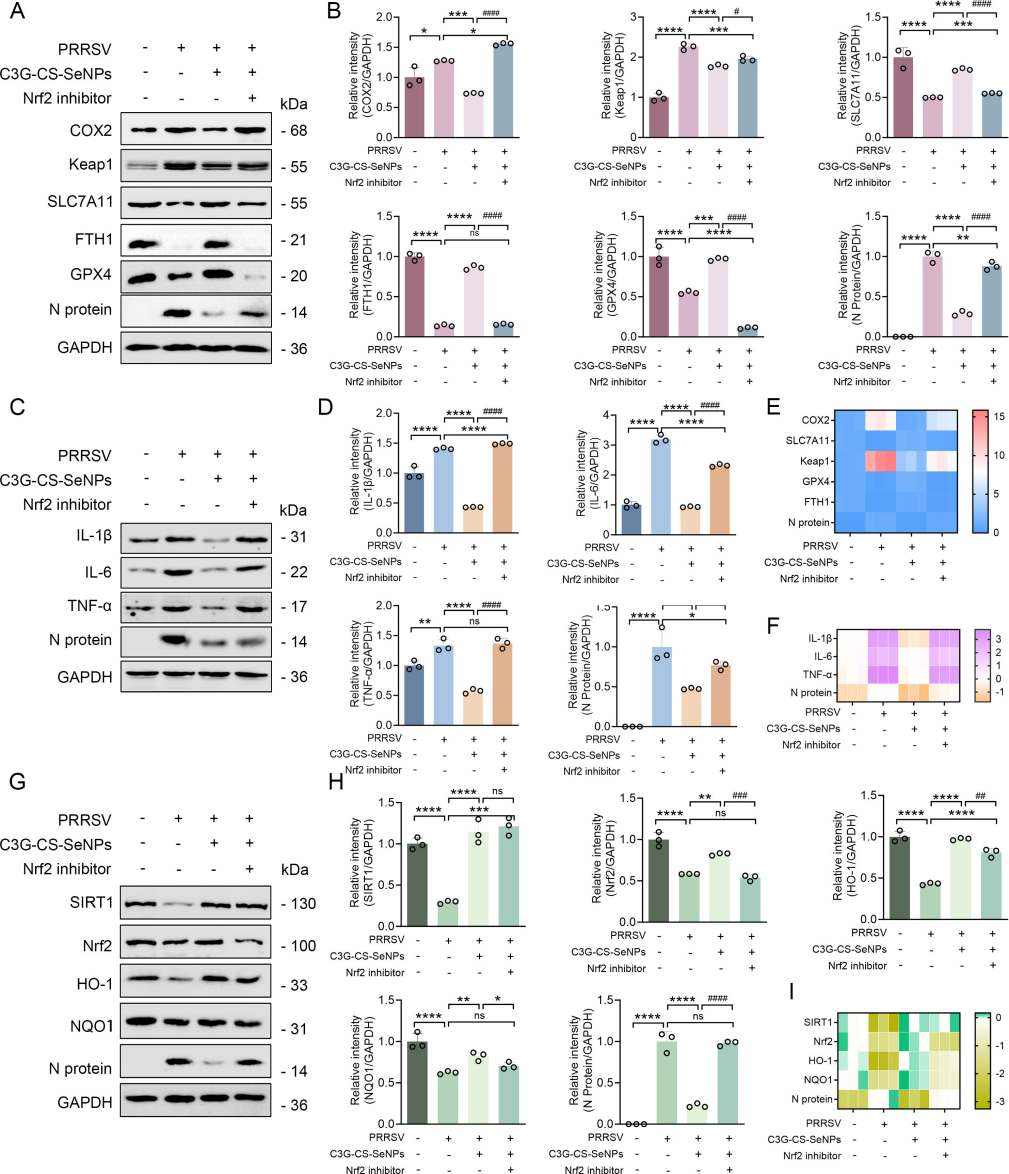
Nrf2 inhibition reverses the protective effects of C3G-Cs-SeNPs by aggravating ferroptosis, inflammation, and oxidative stress. (**A**) Western blot analysis of ferroptosis-related proteins (COX-2, SLC7A11, KEAP1, FTH1, and GPX4) and PRRSV N protein in cells treated with ML385 (Nrf2 inhibitor). (**B**) Quantification of protein bands shown in (**A**) to evaluate relative protein expression. (**C**) Western blot analysis of inflammatory cytokines (IL-1β, IL-6, and TNF-α) following Nrf2 inhibition. (**D**) Quantitative densitometry of inflammatory protein expression in (**C**). (**E**) mRNA expression levels of ferroptosis-related genes (Cox-2, Slc7a11, Keap1, Fth1, and Gpx4) and N gene assessed by qRT-PCR. (**F**) mRNA levels of pro-inflammatory cytokines (Il-1β, Il-6, and Tnf-α) determined by qRT-PCR. (**G**) Western blot analysis of oxidative stress-related signaling proteins (SIRT1, Nrf2, HO-1, and NQO1). (**H**) Quantitative analysis of protein levels in (**G**). (**I**) mRNA expression of SIRT1, Nrf2, Ho-1, and Nqo1. Data are presented as mean ± SD (*n* = 3); *P < 0.05, **P < 0.01, ***P < 0.001 vs. control or indicated groups.

To further establish Nrf2’s functional role in the protective effects of C3G-Cs-SeNPs, we treated cells with the Nrf2 inhibitor ML385. As shown in Fig. 10A, ML385 treatment significantly increased ferroptosis-related markers (COX-2, Keap1), while reducing SLC7A11, GPX4, and FTH1 levels. Concurrently, PRRSV N protein expression rose, indicating heightened viral replication. Quantitative analysis confirmed these trends statistically (Fig. 10B).

Similarly, WB significantly revealed the upregulation of pro-inflammatory cytokines (IL-1β, IL-6, and TNF-α) following Nrf2 inhibition (Fig. 10C), as supported by quantitative analysis (Fig. 10D). RT-qPCR confirmed these findings, showing elevated transcription of COX-2, Keap1, and PRRSV N genes, alongside suppressed SLC7A11*,* GPX4*,* and FTH1 genes (Fig. 10E). Inflammatory cytokine transcripts (IL-1β, IL-6, TNF-α) were similarly enhanced (Fig. 10F).

Furthermore, Nrf2 inhibition also attenuated the expression of antioxidant signaling proteins HO-1 and NQO1, despite unaltered SIRT1 expression (Fig. 10G). These reductions were validated quantitatively (Fig. 10H) and at the transcript level (Fig. 10I). Together, these data demonstrate that Nrf2 activation is critical for C3G-Cs-SeNPs to exert antioxidant and anti-ferroptotic effects via HO-1/NQO1 induction and SLC7A11/GPX4 stabilization. C3G-Cs-SeNPs also mediate anti-inflammatory and antiviral activity by suppressing cytokines and inhibiting PRRSV replication via Nrf2 activation. The reversal of protection upon Nrf2 inhibition underscores the SIRT1/Nrf2 pathway as a central signaling hub mediating these therapeutic outcomes.

### Prospects and limitations of C3G-Cs-SeNPs

The self-assembled C3G-Cs-SeNPs developed in this study demonstrate multifaceted therapeutic potential for treating viral infections like PRRSV and other oxidative stress and inflammation-related diseases. Their ability to modulate the SIRT1/Nrf2/HO-1 pathway suggests broader applicability in diseases where redox imbalance and ferroptosis play critical roles, such as neurodegenerative disorders, ischemic injuries, and chronic inflammatory conditions. Future research could explore their efficacy against other respiratory viruses like influenza, SARS-CoV-2, or non-respiratory conditions. In addition, the scalability of nanoparticle synthesis using natural compounds like C3G and chitosan offers a sustainable and cost-effective platform for large-scale production. Further optimization of nanoparticle pharmacokinetics and biodistribution could enhance targeting specificity and bioavailability. Combining C3G-Cs-SeNPs with existing antiviral agents or immunomodulators could improve therapeutic outcomes.

While this study reveals the therapeutic potential of C3G-Cs-SeNPs, it has some limitations. The study used *in vitro* models, which may not fully capture the complexity of PRRSV pathogenesis in *vivo*. Future work should validate results in porcine models to assess efficacy, safety, and pharmacokinetics. The long-term stability and potential toxicity of C3G-Cs-SeNPs in biological environments need further investigation, particularly regarding selenium accumulation and off-target effects. The precise molecular interactions between C3G-Cs-SeNPs and cellular targets need further elucidation. The nanoparticle’s impact on host immune responses beyond inflammation and ferroptosis requires further investigation.

### Conclusion

**Fig 11:**
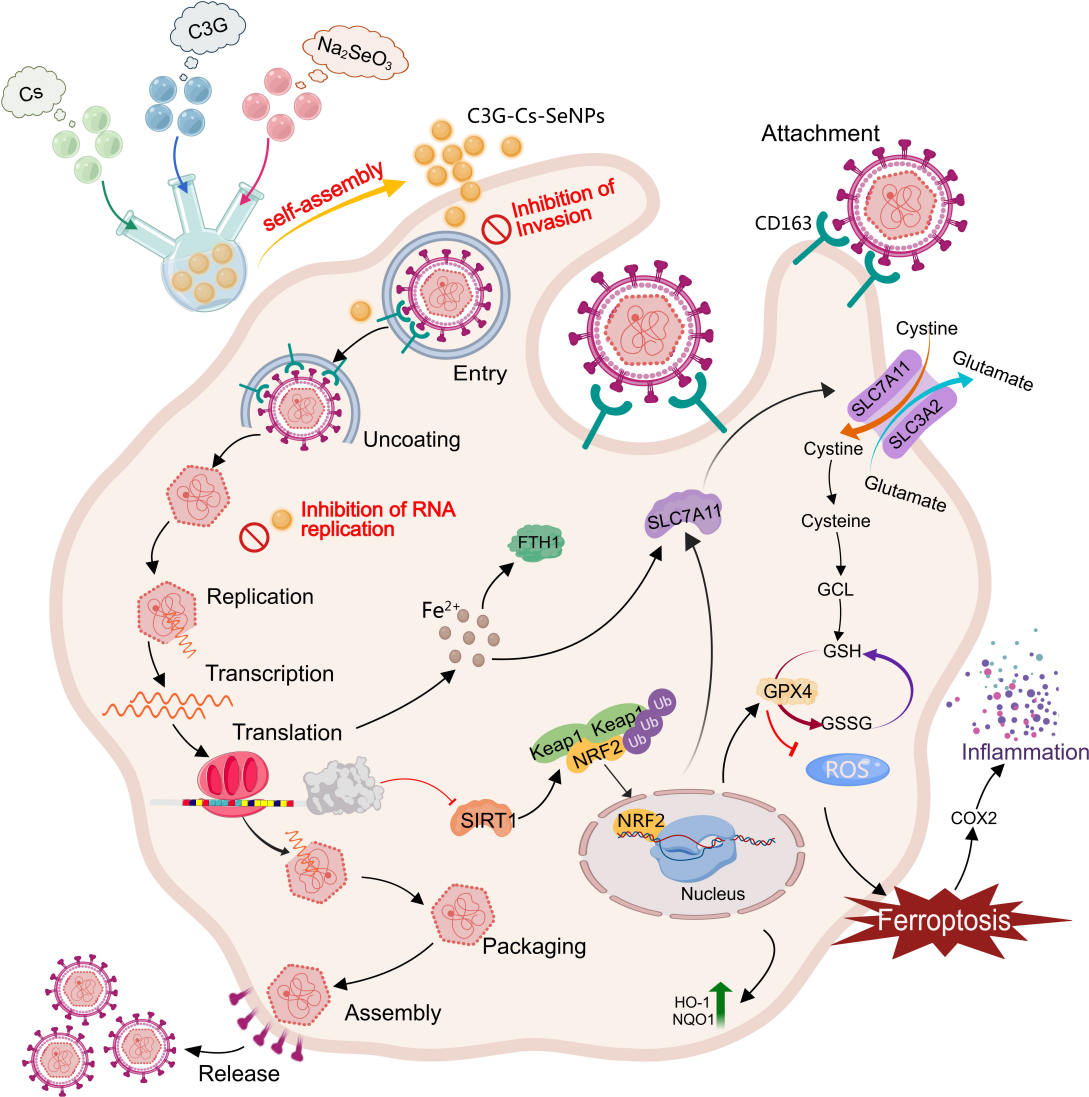
Schematic diagram of C3G-Cs-SeNPs impairs inflammation and ferroptosis induced by PRRSV infection.

Herein, C3G-Cs-SeNPs, synthesized through a self-assembly method, exhibit promising antiviral potential against PRRSV infections. These nanoparticles demonstrate good stability and excellent biocompatibility with minimal cytotoxicity, effectively enhancing antioxidant capacity by reducing ROS in PRRSV-infected cells, thereby mitigating oxidative stress and ferroptosis. Furthermore, C3G-Cs-SeNPs significantly inhibit key stages of the PRRSV lifecycle, including viral internalization and replication, leading to decreased viral proliferation. They also help maintain redox homeostasis by increasing antioxidant enzyme activity. Notably, C3G-Cs-SeNPs may activate the SIRT1/NRF2/HO-1 signaling pathway, which regulates inflammation and ferroptosis in infected cells (Fig. 11). Exploring the crosstalk within this molecular mechanism holds significant theoretical and clinical implications for respiratory infections. The ability of C3G-Cs-SeNPs to simultaneously inhibit viral replication, inflammation, and ferroptosis underscores their translational potential. Unlike vaccines that require strain-specific updates, host-directed therapies like C3G-Cs-SeNPs may offer broad-spectrum protection against PRRSV variants. Thus, C3G-Cs-SeNPs could be a promising therapeutic candidate for preventing and treating inflammation and ferroptosis related to PRRSV infection.

## Data Availability

All data are available within the figures or are available upon request.

## SUPPLEMENTAL MATERIAL

Supplemental material jvi.00954-25-s0001.docx Fig. S1 to S5.

ASM does not own the copyrights to Supplemental Material that may be linked to, or accessed through, an article. The authors have granted ASM a non-exclusive, world-wide license to publish the Supplemental Material files. Please contact the corresponding author directly for reuse.
