# Supplementary material for: Self-assembled cyanidin-3-O-glucoside nanoparticles alleviate inflammation and ferroptosis induced by PRRSV infection
Source: J Virol. 2025 Aug 14;99(9):e00954-25. doi: 10.1128/jvi.00954-25 (PMC12455928; doi:10.1128/jvi.00954-25)
Supplement: Supplemental material — Fig. S1 to S5. [file jvi.00954-25-s0001.docx]

Supplementary Materials for

**Self-assembled Cyanidin-3-O-glucoside Nanoparticles Alleviate Inflammation and Ferroptosis Induced by PRRSV Infection**

Xiaohan Chen^1#^, Yipeng Pang^1#^, Fructueux Modeste Amona^1#^, Zilu Liu^1^, Fang Wang^1^, Yuan Liang^1^, Jiachen Yang^1^, Wanhan Zhang^1^, Xingtang Fang^1^*, Xi Chen^1^*.

^1^ Institute of Cellular and Molecular Biology, School of Life Science, Jiangsu Normal University, Xuzhou, 221116, Jiangsu, China;

^#^These authors contributed equally.

*Corresponding author. Email: [xtfang11@126.com](mailto:xtfang11@126.com) (Xingtang Fang) and [cxvirus@126.com](mailto:cxvirus@126.com) (Xi Chen)

**
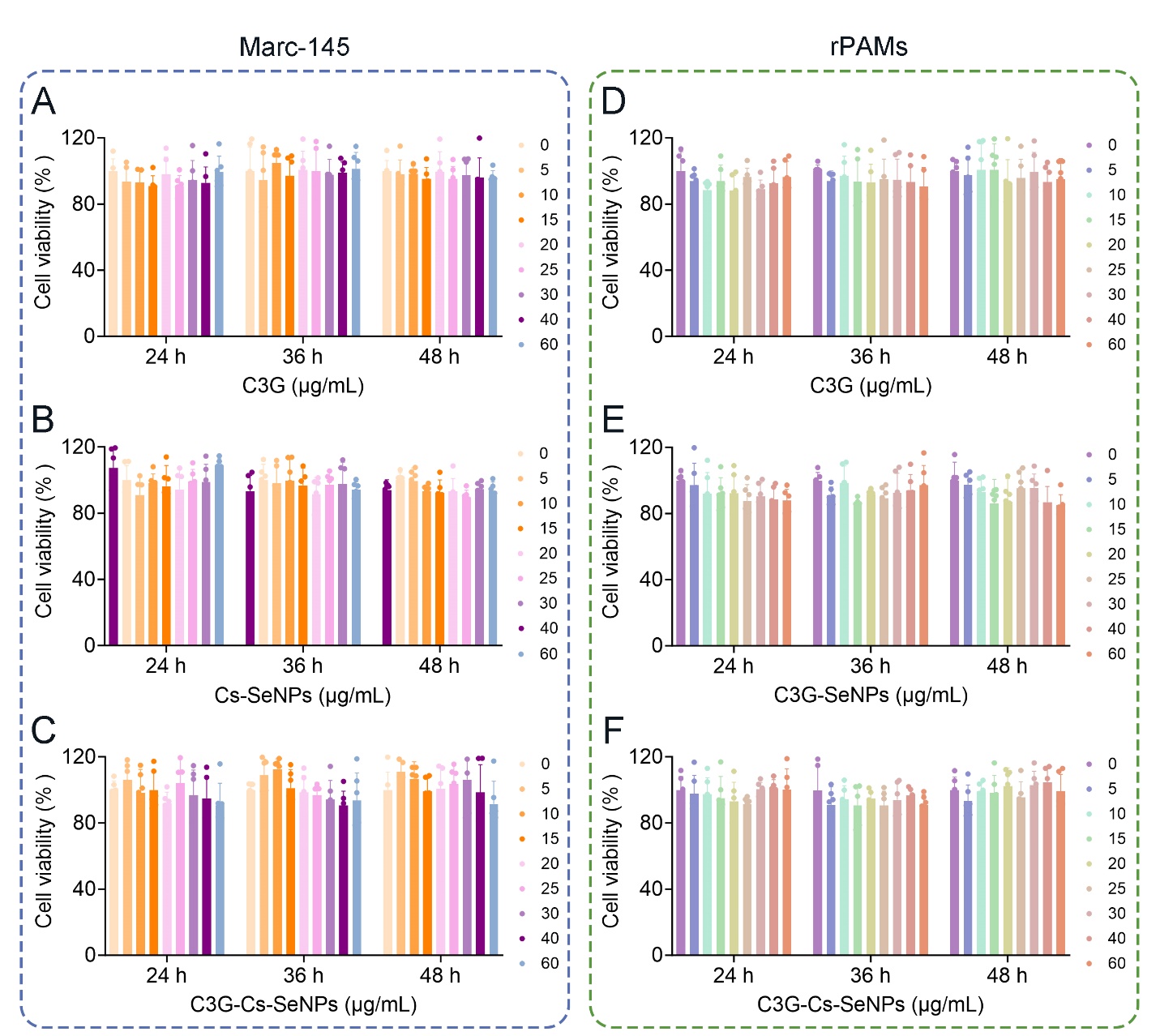
**

**Fig S1. Cytotoxicity of C3G, Cs-SeNPs, and C3G-Cs-SeNPs on Marc-145 cells and rPAMs**. Marc-145 cells and rPAMs were cultured separately with different concentrations of C3G (A, D), Cs-SeNPs (B, E), and C3G-Cs-SeNPs (C, F) (0–60 μg/mL) for 24, 36, and 48 h.


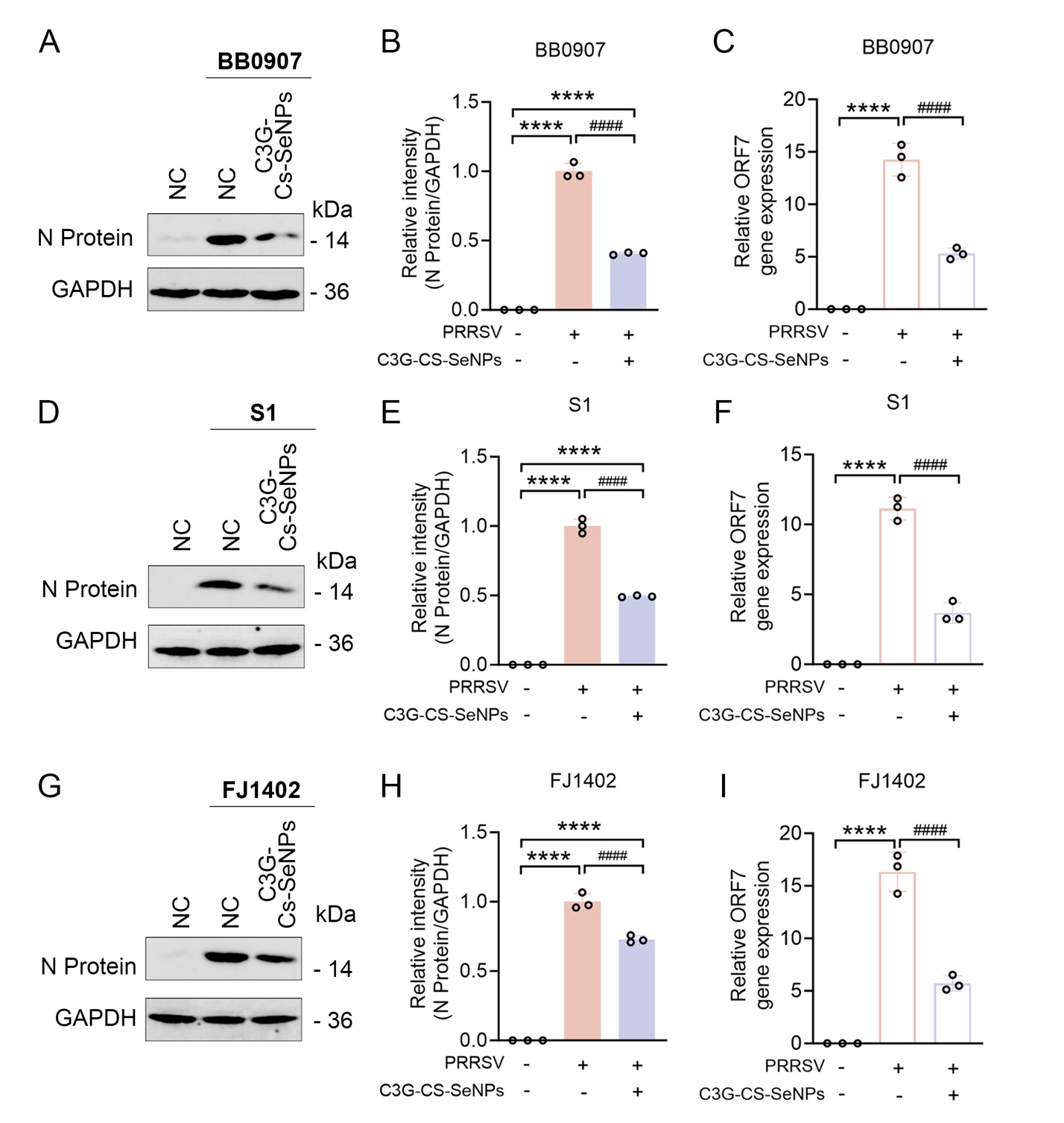


**Figure S2.** **Antiviral activity of C3G-Cs-SeNPs on different PRRSV strains.** (A) Western blot showing N protein levels in BB0907-treated cells. (B) Quantification of N protein expression relative to GAPDH (n=3). (C) ORF7 gene expression measured via qRT-PCR. (D) Western blot of N protein in S1-treated cells. (E) Quantitative analysis of N protein levels (n=3). (F) ORF7 gene expression assessed by qRT-PCR. (G) Western blot for N protein. (H) Quantification of N protein expression (n=3). (I) ORF7 gene expression via qRT-PCR. Results are shown as mean ± SD.*^###^P < 0.001; ^##^P < 0.01; ^#^P < 0.05* vs DMSO-treated cells. ****P < 0.001; **P < 0.01; *P < 0.05* vs. PRRSV-infected rPAMs.


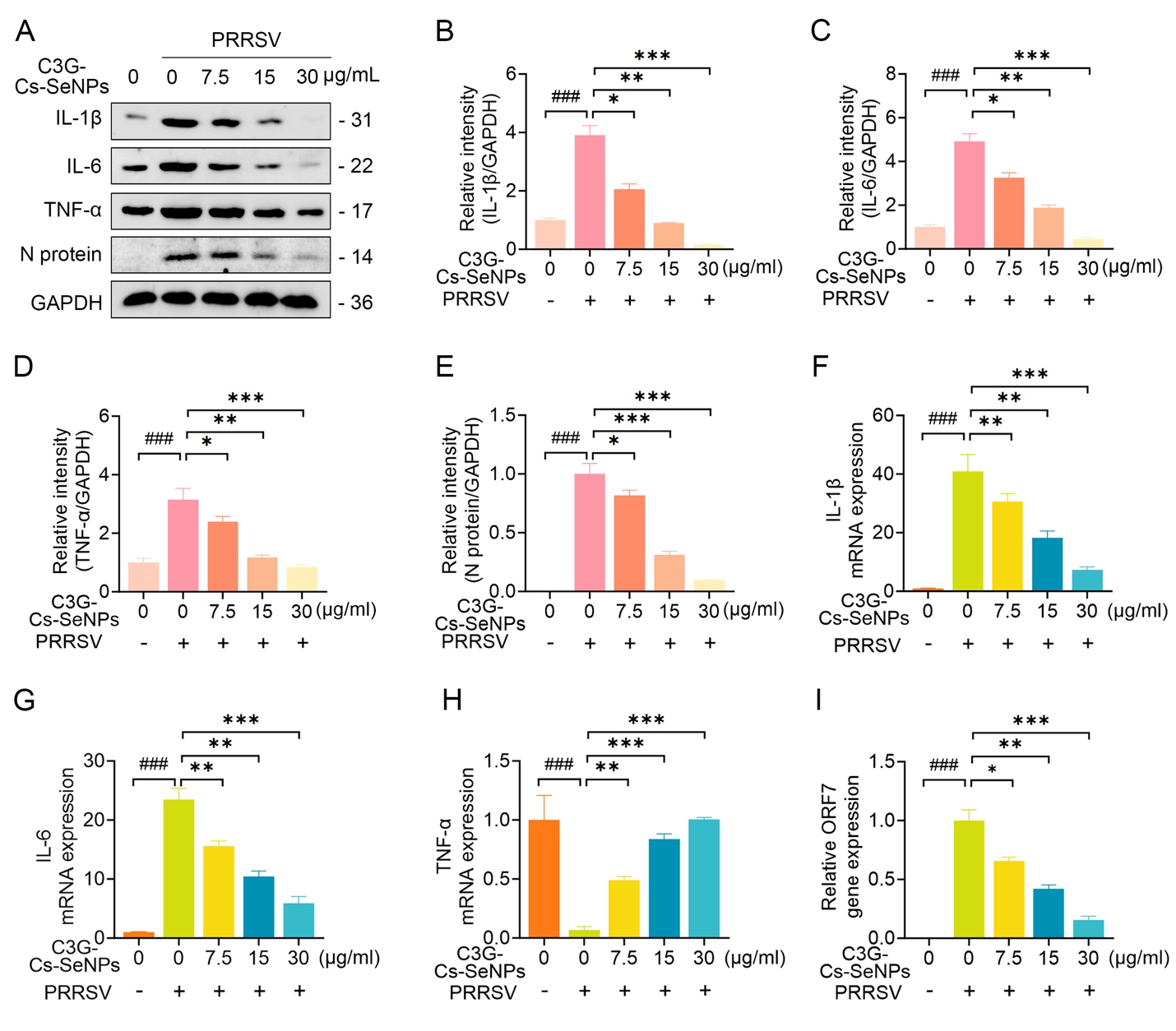


**Fig S3**. **C3G-Cs-SeNPs inhibit inflammation in rPAMs infected by PRRSV.**

rPAMs were treated with PRRSV for 1 hour, followed by treatment with C3G-Cs-SeNPs at different concentrations for 24 hours. (A-E) Western blot analysis showed the effect of C3G-Cs-SeNPs on the protein expression levels of inflammatory cytokines in PRRSV-infected rPAMs. (F-I) RT-qPCR showed the effect of C3G-Cs-SeNPs on mRNA expression of inflammatory factors and N protein. Means ± SD from three independent experiments performed in triplicate.*^###^P < 0.001; ^##^P < 0.01; ^#^P < 0.05* vs DMSO-treated cells. ****P < 0.001; **P < 0.01; *P < 0.05* vs. PRRSV-infected rPAMs.

**
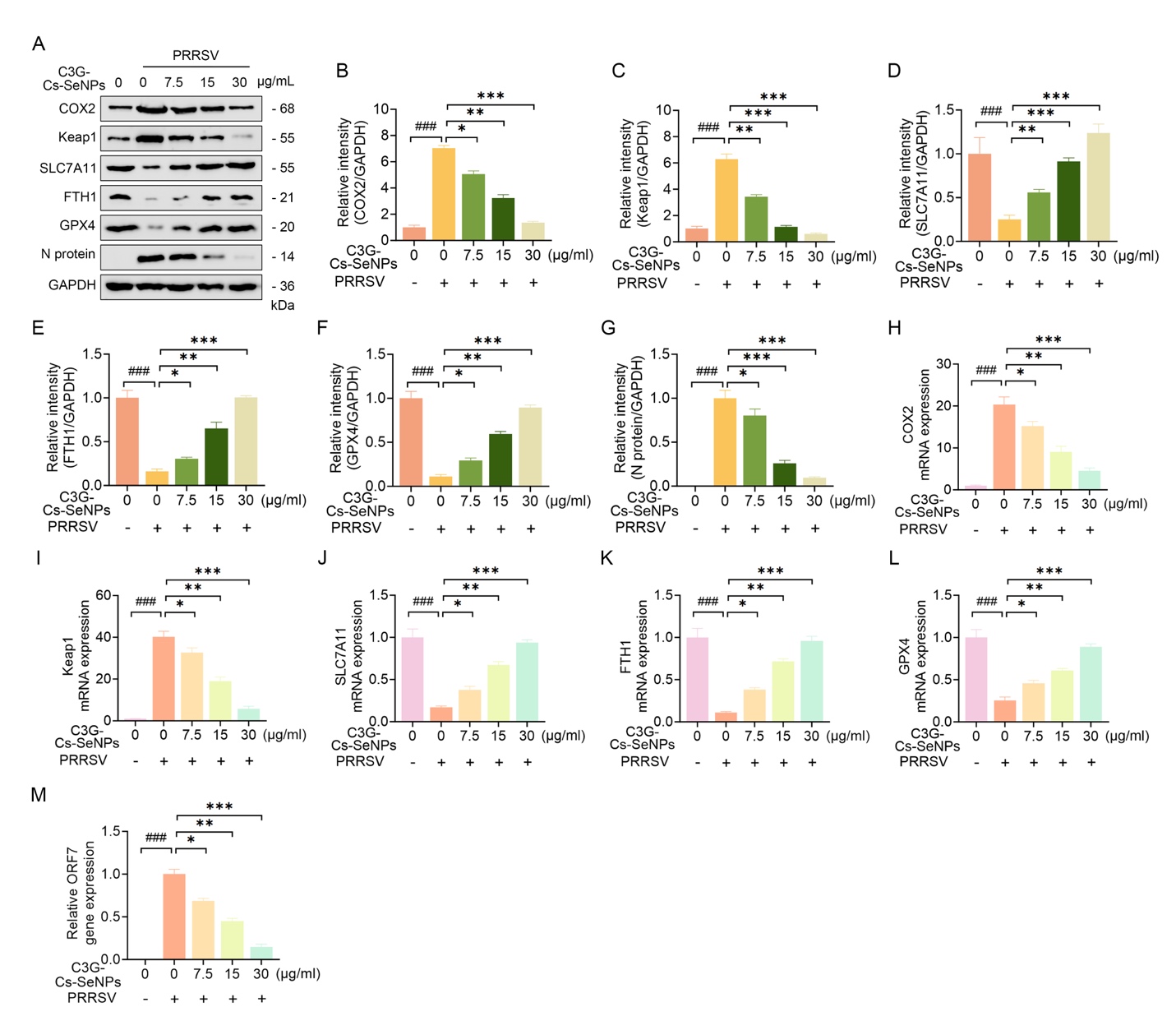
**

**Fig S4**. **C3G-Cs-SeNPs inhibit ferroptosis in PRRSV-infected rPAMs**.

(A-G) Western blot showed the effects of C3G-Cs-SeNPs on the protein expressions of ferroptosis-related markers COX2 (B), Keap1 (C), SLC7A11 (D), FTH1 (E), GPX4 (F), and N protein (G) in PRRSV-infected rPAMs. (H-M) RT-qPCR showed the mRNA expression levels of COX2 (H), Keap1 (I), SLC7A11 (J), FTH1 (K), GPX4 (L), and the ORF7 gene (M). Means ± SD from three independent experiments performed in triplicate. ###P < 0.001; ##P < 0.01; #P < 0.05 vs DMSO-treated cells. ****P < 0.001; **P < 0.01; *P < 0.05* compared with the PRRSV-infected cells.

**
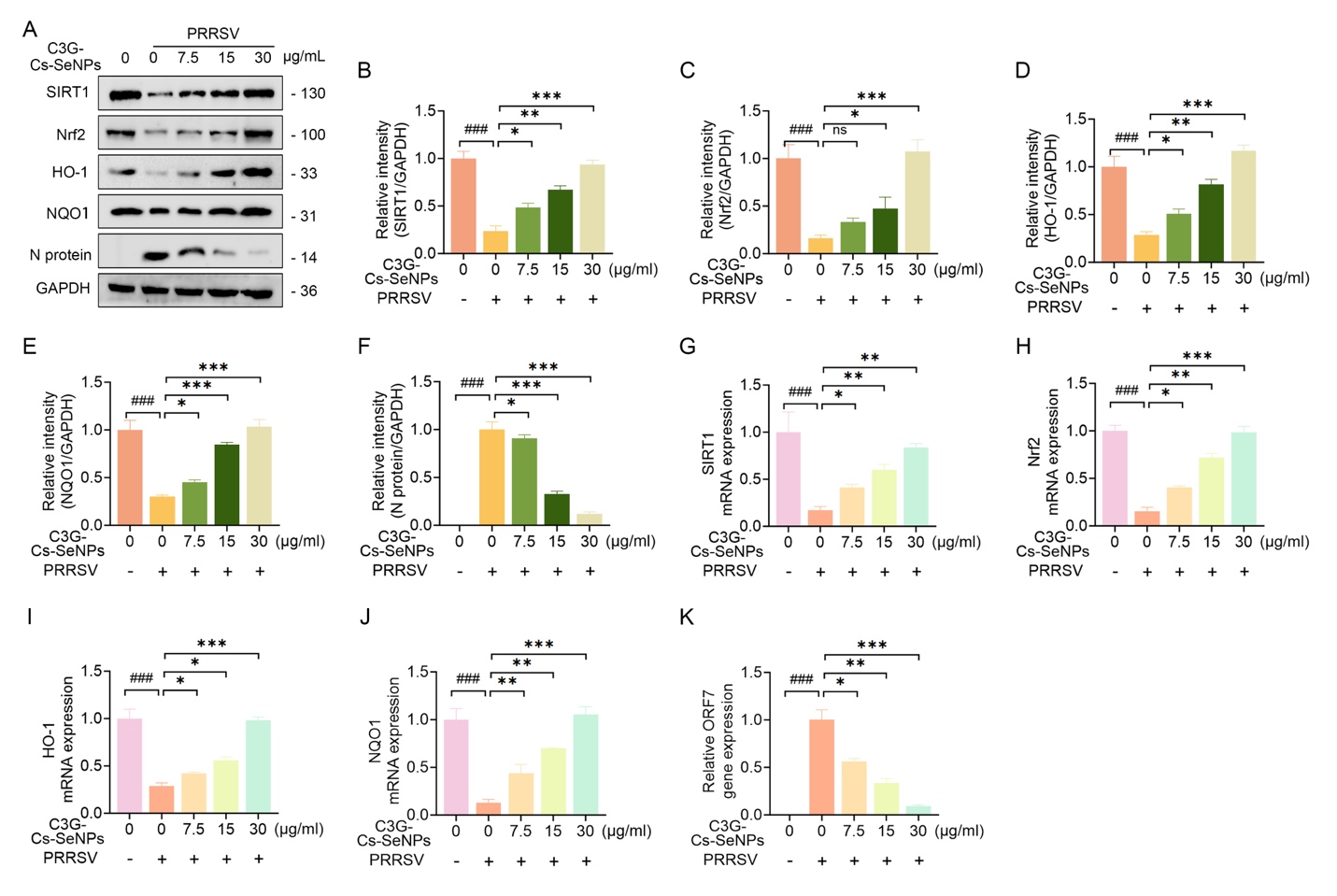
**

**Fig S5. C3G-Cs-SeNPs inhibit inflammation and ferroptosis in rPAMs infected by PRRSV through the SIRT1/Nrf2 signaling pathway.**

(A-F) Western blot analysis showed the effect of C3G-Cs-SeNPs on the protein expressions of the SIRT1/Nrf2 signaling pathway in PRRSV-infected rPAMs. (G-K) RT-qPCR showed the effect of C3G-Cs-SeNPs on mRNA expression of the SIRT1/Nrf2 signaling pathway in PRRSV-infected rPAMs. Means ± SD from three independent experiments performed in triplicate. *^###^P < 0.001; ^##^P < 0.01; ^#^P < 0.05* vs DMSO-treated cells. ****P < 0.001; **P < 0.01; *P < 0.05* vs. the PRRSV-infected cells
